# Motor development in infancy and spine shape in early old age: Findings from a British birth cohort study

**DOI:** 10.1002/jor.24656

**Published:** 2020-03-18

**Authors:** Fiona R. Saunders, Jennifer S. Gregory, Anastasia V. Pavlova, Stella G. Muthuri, Rebecca J. Hardy, Kathryn R. Martin, Rebecca J. Barr, Judith E. Adams, Diana Kuh, Richard M. Aspden, Rachel Cooper, Alex Ireland

**Affiliations:** ^1^ School of Medicine, Medical Sciences and Nutrition, Aberdeen Centre for Arthritis and Musculoskeletal Health, Institute of Medical Sciences University of Aberdeen Aberdeen UK; ^2^ School of Health Sciences Robert Gordon University Aberdeen UK; ^3^ MRC Unit for Lifelong Health and Ageing at UCL London UK; ^4^ Cohort and Longitudinal Studies Enhancement Resources (CLOSER) UCL Institute of Education London UK; ^5^ Medicines Monitoring Unit (MEMO), School of Medicine, Division of Molecular and Clinical Medicine, Ninewells Hospital and Medical School University of Dundee Dundee UK; ^6^ Manchester Academic Health Science Centre and Radiology, Manchester Royal Infirmary Central Manchester University Hospitals NHS Foundation Trust and University of Manchester Manchester UK; ^7^ Department of Sport and Exercise Sciences, Research Centre for Musculoskeletal Science and Sports Medicine Manchester Metropolitan University Manchester UK; ^8^ Department of Life Sciences, Research Centre for Musculoskeletal Science and Sports Medicine Manchester Metropolitan University Manchester UK

**Keywords:** growth, loading, mechano‐adaptation

## Abstract

Spine shape changes dramatically in early life, influenced by attainment of developmental milestones such as independent walking. Whether these associations persist across life is unknown. Therefore, we investigated associations between developmental milestones and spine shape, as determined using statistical shape models (SSMs) of lumbar spine from dual‐energy X‐ray absorptiometry scans in 1327 individuals (688 female) at 60 to 64 years in the MRC National Survey of Health and Development. Lumbar lordosis angle (L4 inferior endplate to T12 superior endplate) was measured using the two‐line Cobb method. In analyses adjusted for sex, height, lean and fat mass, socioeconomic position, and birthweight, later walking age was associated with greater lordosis described by SSM1 (regression coefficient, 0.023; 95% CI, 0.000‐0.047; *P* = .05) and direct angle measurement. Modest associations between walking age and less variation in anterior‐posterior vertebral size caudally (SSM6) were also observed (0.021; 95% CI, −0.002 to 0.044; *P* = .07). Sex interactions showed that later walking was associated with larger relative vertebral anterior‐posterior dimensions in men (SSM3; −0.043; 95% CI, −0.075 to 0.01; *P* = .01) but not women (0.018; 95% CI, −0.0007 to 0.043; *P* = .17). Similar associations were observed between age at independent standing and SSMs but there was little evidence of association between sitting age and spine shape. Unadjusted associations between walking age and SSMs 1 and 6 remained similar after adjustment for potential confounders and mediators. This suggests that these associations may be explained by altered mechanical loading of the spine during childhood growth, although other factors could contribute. Early life motor development, particularly walking, may have a lasting effect on the features of spine morphology with clinical significance.

## 1 INTRODUCTION

Infancy and early childhood represent key periods for the development of spine shape and structure. Lordosis (indicated by the lumbosacral angle) increases from 20° to 70° in the first 5 years of life,^1^ followed by slower growth in both lordosis and thoracic kyphosis up to adulthood.^2^ In contrast, cervical lordosis increases until 9 to 10 years of age before decreasing throughout adolescence.^3^ Vertebral height and width increase dramatically in the first 2 years of life, after which time more modest growth continues until adulthood.^4^ These growth patterns are highly dependent on vertebral location, with greater growth in lumbar than thoracic and in turn cervical bodies^4^ in line with the loading they experience. Due to these increases in both vertebral size and bone mineral density, lumbar spine bone mass increases fivefold between the ages of 1 and 36 months.^5^

A key factor in the development of spine shape during this period is attainment of motor milestones at 6 to 24 months of age. This development coincides with a large increase in lordosis, and this angle is closely associated with stages of motor development such as standing, walking, and running.^1^ The influence of early life motor development on spine shape can also be examined through comparison with groups where attainment of motor skills is impaired. Children with cerebral palsy display impaired growth of vertebral bodies, with these deficits emerging after typical walking age at around 2 years.^4^ In children with osteogenesis imperfecta, earlier attainment of independent sitting is associated with delayed development of scoliosis.^6^ However, it is unknown whether associations between early life motor development and spine shape persist into adulthood.

Development of spine shape involves simultaneous but discordant regional changes in vertebral size and shape, as well as overall curvature.^7^, ^8^ Studies of spine shape have typically described only a small number of these variables. Statistical shape modeling (SSM) can provide an objective description of variation in these and other aspects of spine shape (such as degree of variation in vertebral size within an individual's spine). SSM has been shown to be more reliable and accurate than traditional measurements of spinal curvature.^9^, ^10^

Therefore, our primary aim was to examine whether early childhood motor development, as indicated by age of attainment of independent walking is associated with spine shape in old age using data from the MRC National Survey of Health and Development (NSHD), a British birth cohort study. Walking age was selected as the motor milestone of primary interest because of the large loads experienced during this movement^11^ and previous reports of strong associations between walking age and bone health throughout life.^12^, ^13^, ^14^ While spine shape was our primary outcome, as a secondary aim we also examined associations between walking age and osteoarthritis of the spine to assess whether there was any evidence that our main findings have clinical consequences that are detectable in early old age. As age at attainment of sitting and standing have also been associated with skeletal development,^1^, ^6^ and are highly correlated with age at walking, associations between these exposures and spine shape were also assessed as secondary analyses. It was hypothesized that the age at which independent walking was attained would be associated with variation in spine shape features in early old age.

## 2 METHODS

### 2.1 Study population

The NSHD is a birth cohort study consisting of a socially stratified sample of 5362 singleton births in 1 week in March 1946 in England, Scotland, and Wales. These participants have been prospectively followed regularly since birth.^15^ Between 2006 and 2010, eligible participants known to be alive and living in England, Scotland, and Wales were invited for an assessment at one of the six clinical research facilities (CRF). Of the 2856 individuals invited, 1690 attended a CRF and 539 received a home visit from a research nurse. Ethical approval for this data collection was obtained from the Central Manchester Research Ethics Committee (07/H1008/245) and the Scottish A Research Ethics Committee (08/MRE00/12).

### 2.2 Spine DXA images

During the CRF assessment, images of the total body and spine were obtained using a QDR 4500 Discovery dual‐energy X‐ray absorptiometry (DXA) scanner (Hologic Inc, Bedford, MA). In five centers, scanners had rotating C‐arms allowing participants to lie supine for all scans, while one center used a scanner with a fixed C‐arm requiring participants to be scanned in a lateral decubitus position. In both cases, participants were scanned with hips and knees flexed, and with arms raised so as not to obscure the scanned region. Judith E. Adams' laboratory performed quantitative analysis of all scans and assessments for image quality. A manufacturer‐provided phantom was scanned daily before participant scanning; once a month, these results were sent to the coordinating center for scrutiny.

### 2.3 Statistical shape modeling

Of the 1690 participants who attended a CRF, 1601 had a spine DXA scan. Seventy‐two images were excluded from analysis: in 41 images vertebral outlines could not be clearly determined, 23 had scanning artifacts, 5 did not include all vertebrae of interest, 2 included metalwork, and excessive axial rotation was observed in 1 image. This left 1529 images which were used to build the SSM; this process has been described in detail previously.^16^ Briefly, custom‐made Shape software (University of Aberdeen) was used to create a template of 89 points including all vertebrae from the 10th thoracic vertebra (T10) to the superior endplate of the 5th lumbar vertebrae. These eight vertebrae were chosen for analysis as they were visible on all scans. Following an automatic search and placement of points, all images were manually checked and where necessary points were adjusted. Mean intra‐ and inter‐rater repeatability for this technique is 1.4 and 2.2 pixels, respectively,^16^ which represents a small error considering an average spine image size of 1200 × 400 pixels and a typical vertebra size of approximately 80 × 60 pixels. Procrustes transformation was used to translate, rotate, and scale the images to remove influences of size and alignment. Principal component analysis was then performed to generate independent orthogonal modes of variation, describing in descending order of percentage variation standardized to a mean of 0 and standard deviation of 1. Eight modes (SM1 to SM8) were identified which each accounted for greater than 1% spine shape variation ranging from SM1 which accounted for 53.0% of variation to SM8 which accounted for 1.2%; in total, these eight modes accounted for 84.9% of the total variance.^16^ Lumbar lordosis angle was measured using the two‐line Cobb method^17^ between the inferior endplate of L4 and the superior endplate of T12. For each endplate we used the SSM point coordinates for the vertebral “corners” to plot a line and calculate the slope of that line. Using custom‐written code in MATLAB (R2018a; The Mathworks, Natick, MA) the angle of intersection of the two lines was calculated in degrees for each image in the dataset.

### 2.4 Age at onset of independent walking

The age in months at which their child first walked unaided was recalled by participants' mothers during an assessment at age 2 years.

### 2.5 Covariates

Potential confounders and mediators of the main associations between walking age and each spine shape mode were selected a priori based on existing literature.^12^, ^16^, ^18^ The potential confounders were birthweight, childhood socioeconomic position (SEP), adult SEP, and height, and the potential mediators were appendicular lean mass and appendicular fat mass. Birthweight was extracted from medical records within a few days of birth, and measurements to the nearest quarter‐pound (113 g) were converted to kilograms. As indicators of SEP, father's occupation at age 4 years (or at age 11 or 15 if missing at age 4) and own occupation at age 53 years (or if not available, the most recent measure in adulthood) were both categorized into six groups (I [professional], II [managerial and technical], IIINM [skilled nonmanual], IIIM [skilled manual], IV [partly skilled], and V [unskilled]) using the Registrar General's Social Classification.^19^ During the CRF visit, height was measured to the nearest millimeter and recorded in centimeters, and appendicular lean and fat mass in kilograms were estimated from total body DXA scans.

### 2.6 Statistical analysis

We include 1327 participants (688 women) in our models. Of the 1529 participants with spine shape mode data, 106 had missing data on age of independent walking and a further 96 had missing data on covariates. Complete case analysis was undertaken using the R statistical environment (version 3.2.2; www.r‐project.org). Associations between age at onset of independent walking and each spine shape mode were assessed using multiple linear regression models. There was no evidence of deviation from linearity when quadratic terms were included, so walking age was modeled as a continuous linear variable. Walking age × sex interactions were examined given previous findings of sex‐specific associations of walking age with bone outcomes.^12^ Where sex interactions were identified (*P* < .1), subsequent models were sex‐stratified. Model 1 was adjusted for sex (unless sex‐stratified) and CRF location (as one CRF used a scanner with a fixed C‐arm requiring participants to be moved between scans). The impact of adjustment for each of the confounders and mediators identified above was then examined in turn before all covariates were entered into a final model (model 2) simultaneously. Associations between walking age and lumbar lordosis angle were assessed using the same model structures.

In addition to describing associations between walking age and individual mode scores, we wanted to examine how overall spine shape described by these modes varied between earlier and later walkers. Therefore, we combined mean mode scores for early walkers (defined as −2 SD below the mean walking age [ie, 9.0 months]) and late walkers (defined as +2 SD above the mean walking age [ie, 18.5 months]) for both women and men to generate mean spine shapes.

### 2.7 Sensitivity analyses

While the prevalence of radiographic spine osteoarthritis in the NSHD cohort is low, we investigated whether there were any associations between walking age and osteoarthritis of the spine at age 60 to 64 years. DXA images were graded using a validated atlas scoring system,^20^ with grades of 0 to 3 for each vertebra (T10‐L4) summed to give a total Lane grade (TLG). We also assessed associations between sitting (mean, 6.5 ± 1.4 months) and standing age (mean, 11.4 ± 2.1 months) obtained at the same maternal interview as walking age and spine shape modes using models described above.

## 3 RESULTS

Characteristics of the participants in this study are detailed in Table 1 and spine shapes described by each mode are presented in Figure S1. Scores for SM1, SM3, and SM8 were greater in women than men, whereas men had a higher score for SM6. Lumbar lordosis angle was also greater in women than men.

**Table 1:** Characteristics of the MRC National Survey of Health and Development stratified by sex (sample restricted to those with complete spine shape mode data and covariates)

	Women (n = 688)		Men (n = 639)		
Variable	Mean	SD	Mean	SD	Sex difference, *P* value
Walking age, mo	13.7	2.4	13.7	2.3	.6
Birthweight, kg	3.39	0.63	3.45	0.57	.05

	***n***	**%**	***n***	**%**	
Father's occupational class (age, 4 y)					
I	51	7.4	58	9.1	.72
II	158	23.0	141	22.1	
IIINM	130	18.9	125	19.6	
IIIM	195	28.3	184	28.8	
IV	122	17.7	97	15.2	
V	32	4.7	34	5.3	
Own occupational class (age, 53 y)					
I	14	2.0	86	13.5	<.01
II	293	42.6	303	47.4	
IIINM	246	35.8	71	11.1	
IIIM	39	5.7	136	21.3	
IV	73	10.6	35	5.5	
V	23	3.3	8	1.3	

**Musculoskeletal assessments at 60‐64 y**	**Mean**	**SD**	**Mean**	**SD**	
Age at time of assessment, y	63.2	1.1	63.1	1.2	.09
Height, m	1.62	0.06	1.75	0.06	<.01
Weight, kg	71.4	12.3	84.9	12.6	<.01
Appendicular lean mass, kg	16.1	2.4	24.6	3.3	<.01
Appendicular fat mass, kg	14.3	4.1	10.0	2.8	<.01
Lumbar lordosis angle, °	13.1	7.7	11.5	7.2	<.01
Spine shape mode (SM) scores					
SM1	0.07	1.02	−0.05	0.96	.03
SM2	−0.03	0.99	0	1	.62
SM3	0.47	0.78	−0.49	0.98	<.01
SM4	−0.05	1.02	0.03	0.98	.12
SM5	−0.04	0.97	0.05	1	.1
SM6	−0.18	0.98	0.19	0.96	<.01
SM7	−0.01	0.96	0.05	1.03	.28
SM8	0.23	0.92	−0.27	1	<.01

Later age at onset of independent walking was weakly associated with greater SM1 scores in model 1 (regression coefficient, 0.019; 95% CI, −0.004 to 0.041), this association was strengthened in fully adjusted model 2 (0.023; 95% CI, 0.000‐0.047). This suggests that associations in model 1 were obscured by negative confounding, although further analyses of individual factors suggested that this was not attributable to any one single covariate (Table 2).

**Table 2:** Associations between age at onset of independent walking and spine shape mode outcomes in the MRC National Survey of Health and Development

Mode	Group	Model	Regression coefficient	95% CI		*P*	Sex interaction, *P*
SM1	Combined	1	0.019	−0.004	0.041	.1	.28
		2	0.023	0.000	0.047	.05	.36
SM2	Combined	1	−0.002	−0.025	0.021	.88	.76
		2	−0.014	−0.037	0.010	.25	.79
SM3	Men	1	−0.024	−0.056	0.008	.15	.03
	Women		0.021	−0.004	0.046	.09	
	Men	2	−0.043	−0.075	−0.010	.01	<.01
	Women		0.018	−0.007	0.043	.17	
SM4	Combined	1	−0.013	−0.037	0.010	.25	.14
		2	−0.006	−0.030	0.017	.6	.14
SM5	Combined	1	−0.011	−0.034	0.012	.35	.34
		2	−0.017	−0.040	0.007	.17	.45
SM6	Combined	1	0.021	−0.002	0.043	.07	.94
		2	0.021	−0.002	0.044	.07	.91
SM7	Combined	1	0.007	−0.016	0.030	.54	.56
		2	0.006	−0.018	0.030	.63	.39
SM8	Combined	1	0.002	−0.020	0.024	.84	.19
		2	0.010	−0.012	0.033	.36	.24

*Note:* Regression coefficients are the difference in mean SM score per 1 month increase in walking age. Where sex interactions were evident (*P* for interaction <.1), sex‐specific associations are presented. Model 1 adjusted for sex (if men and women are combined) and clinic, model 2: model 1 + birthweight + father's occupational class + adult occupational class + height + appendicular fat mass + appendicular lean mass. Only results from basic and fully adjusted models are presented for brevity. When each set of covariates were adjusted for, in turn, there was no evidence that any one specific set of factors was responsible for the attenuations observed between the models shown here.

There was some evidence to suggest that later walking age was also weakly associated with greater SM6 scores in model 1 (0.021; 95% CI, −0.002 to 0.043); this association was similar in model 2. Sex interactions were evident for SM3 in model 1, with later walking age modestly associated with lower scores in men and higher scores in women. In model 2, the interaction was stronger due to a strengthening of the negative association in males. There was no evidence of associations between walking age and SM2, 4, 5, 7, or 8.

**Figure 1:**
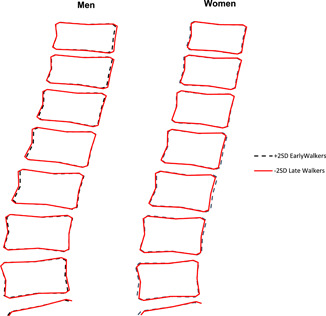
Mean spine shapes described by statistical shape models in early (−2 SD of the mean age) and late‐walking (+2 SD of the mean age) men and women. The mean age of walking in this cohort was 13.7 ± 2.3 months with no sex difference. Therefore, early and late walking as described above corresponded to walking at 9.0 months and walking at 18.5 months, respectively [Color figure can be viewed at wileyonlinelibrary.com]

When taking findings for SM1, 3, and 6 together, in later walkers these modes describe greater lumbar and thoracic lordosis (SM1), and more uniform anterior‐posterior vertebral body diameter relative to vertebral height throughout the spine (SM6). Sex interactions in SM3 indicated greater relative anterior‐posterior vertebral size in late‐walking men but not women. In support of the features described by associations with SM1, walking age was also associated with greater lumbar angle corresponding to an increase in lordosis of 0.57° (95% CI, 0.36° to 0.78°; *P* = .007) for every 1 SD (around 2 months) increase in walking age in model 2. Mean spine shapes generated for early and late‐walking men and women are shown in Figure 1.

### 3.1 Sensitivity analyses

Prevalence and severity of radiographic OA was low in this cohort; 301 individuals (23%) had no evidence of degeneration (grade 0) at any vertebrae, and 898 individuals (68%) had only mild degeneration (grade ≤ 1) at any vertebrae. No associations were observed between walking age and TLG when the latter was modeled either as a continuous or dichotomous variable (based on an TLG > 0 as cut‐off) (*P* > .4 in both cases). Sitting age was weakly positively associated with walking age (*r* ^2^ = .18; *P* < .001), and was weakly negatively associated with spine shape mode 5 only (Table S1; *P* = .06). There was a strong positive association between standing age and walking age (*r* ^2^ = 0.64; *P* < .001). Standing age was weakly positively associated with SM1 scores in model 2 only (regression coefficient, 0.022; 95% CI, −0.004 to 0.047), and with SM6 scores in both models. There was evidence of sex interactions for SM3 with later standing age associated with lower scores in men and higher scores in women, and for SM5 with later standing age associated with lower scores in women only.

## 4 DISCUSSION

The aim of this study was to investigate associations between early life motor development and components of spine shape described by SSMs in early old age. In fully adjusted models, later walking age was modestly associated with greater lordosis and more even vertebral size along the spine, and with greater relative vertebral size in men but not women. Similar associations were observed for later standing age but not for sitting age.

### 4.1 Comparison with previous findings

To our knowledge, this is the first study to investigate associations between early life motor development and spine shape in adulthood. Previous studies have shown associations between attainment of motor development milestones and lordosis in early childhood.^1^ Impaired or delayed motor development has previously been shown to be associated with spine development. Children with cerebral palsy are at risk of developing excessive lordosis of the lumbar spine,^21^ similar to observations of greater lordosis in late walkers in this study. We have previously reported associations between walking age and spine area in males only in this cohort,^12^ which would initially seem to contradict findings of smaller vertebral size in males in this study. However, as can be seen in Figure 1 and Figure S1 these differences are subtle and unlikely to have a substantial influence on overall vertebral area. More importantly, images are scaled before generation of shape modes thereby removing differences in overall size. Greater vertebral size in SM6 therefore represents the relative anterior‐posterior to cranial‐caudal proportions of vertebral bodies, which could result from narrower and/or taller vertebrae. As walking age is positively associated with height in this cohort, greater vertebral height could explain these apparently conflicting associations. Similar associations to those observed between walking age and spine shape were observed for standing age, which was highly correlated with walking age, but there was little evidence of associations between sitting age and spine shape. This is similar to findings of previous studies in younger children, where walking age but not crawling or standing age was associated with tibia mass and geometry.^14^

### 4.2 Possible explanation of findings

Walking is associated with lumbosacral loads of around 1.6 times bodyweight, which is 60% greater than those achieved during standing.^11^ Therefore, attainment of independent walking exposes the spine to large increases in loading at a time of rapid development. The smaller loads associated with static activities may explain the lack of association between sitting age and spine shape. The importance of larger locomotory loads for spine health is supported by the large bone losses associated with loss of ambulation such as in long‐term spaceflight.^22^ Initially, vertebral size is similar throughout the spine^23^ but differences between lumbar and thoracic vertebrae emerge around the onset of walking.^4^ Reduced variation in relative vertebral size in late walkers may, therefore, reflect reduced loading variation throughout the spine during this period. Greater back extensor muscle size has previously been associated with greater lumbar lordosis,^24^ but there was little evidence of associations between lean mass and spine shape modes in this study, and adjustment for lean mass did not attenuate association between walking age and spine shape modes.

### 4.3 Significance and implications

Greater lumbar lordosis, described by SM1 and the lumbar angle, identified in late walkers in the current study have been shown to be associated with spondylolysis and isthmic spondylolisthesis in other studies.^25^ A recent study also found that smaller relative anterior‐posterior size, observed in late‐walking women in this study, was also associated with spondylolysis.^26^ A number of clinical groups with delayed ambulation including Down syndrome,^27^ osteogenesis imperfecta,^28^ and dyskinetic cerebral palsy^29^ have increased risk of spondylolysis and/or isthmic spondylolisthesis, therefore motor deficits in early life may contribute to these problems. If delayed motor development is shown to influence spondylolysis risk in late walkers, there may be interventional opportunities to minimize these effects. Parent‐led walking training can lead to earlier walking onset in the general population^30^ and clinical cohorts such as children with Down syndrome.^31^ In children with myelomeningocele, these interventions appear effective in reducing deficits in bone mass.^32^ While the effects of walking training on joint shape are unknown, future interventional studies investigating these effects could establish motor development as a modifiable factor influencing lifelong spine health. There is conflicting evidence as to whether lumbar lordosis is associated with other types of lower back pain and osteoarthritis,^25^ but these associations were not found in the cohort examined in this study.^33^ We found no evidence of associations between walking age and radiographic OA, although the incidence of OA was very low in this cohort. In addition, to our knowledge there have been no previous investigations of associations between the reduced curvature in the lower thoracic region observed in late walkers in this study and clinical outcomes. Previous observations of lower bone mass in male late walkers in this cohort^12^ suggest an increased risk of fracture, but it is not clear whether spine shape features identified in the current study could influence this risk. Future studies examining associations between motor development and spine pathologies could help reveal the clinical consequences of delayed attainment of motor milestones.

### 4.4 Strengths and weaknesses

The cohort examined in this study is broadly representative of the British‐born population of the same age,^34^ which allows us to generalize these results to this population. In addition, the cohort have been followed for over six decades since birth, allowing us to adjust for potential confounders which were obtained prospectively. Most importantly, details of early life motor development were obtained six decades previously by maternal recall at 2 years, which has been shown to be highly reliable.^35^, ^36^ Previous studies have shown that associations between early life motor development and adolescent bone outcomes are mediated by childhood physical activity.^12^ Due to limited information on physical activity in early life, we were unable to explore this potential mediating pathway, although walking age is not associated with adult physical activity in this cohort.^37^ As an observational study, we cannot attribute causality, and residual confounding and bias due to drop out and missing data in this cohort^38^ may have influenced the results. Caution is required in interpreting these findings and considering their implications, because evidence suggests that some of the associations we have observed in this study are modest. In addition, the overall shape differences between early and late walkers described by SSMs are quite subtle. However, even these small differences (0.2‐0.4 SD) are similar to those identified between individuals with and without long‐term back pain in the same cohort^33^ suggesting that they may prove to be clinically relevant with increasing age. Spine images were taken with participants in a supine position with hips and knees flexed which would result in differences in spine morphology compared with standing. However, we have shown previously that interindividual variation in spine shape is preserved throughout a full range of extension to flexion and in a range of postures,^39^, ^40^ therefore, the current results likely reflect spine shape variation independent of the position. In addition, we could only measure down to inferior endplate of vertebra L4 as the inferior endplate of vertebra L5 was not consistently visible, so measures of lumbar angle from T12 to L4 are surrogate measures of the full lumbar lordosis angle.

## 5 CONCLUSIONS

Later age at onset of independent walking in early childhood is modestly associated with features of spine shape in early old age, namely with greater lordosis and less variation in vertebral size along the spine, and relative vertebral size is greater in male later walkers but not females. These associations were also observed with standing but not sitting age and were independent of a number of potential confounders and mediators, which suggests that they could result from altered mechanical loading during a key phase of growth in early childhood. Clinically, greater lumbar lordosis and smaller vertebral size are associated with spondylolysis and isthmic spondylolisthesis and a number of clinical populations with delayed motor development have greater incidence of these conditions. Early life motor development, in particular, walking onset age, appears to have a small persisting effect on the features of spine morphology with clinical relevance throughout life. Given that training interventions can promote earlier walking onset, age at onset of independent walking may represent a novel modifiable factor to improve spine development particularly in populations in which delayed motor development and spine problems are common.

## CONFLICT OF INTERESTS

The authors declare that there are no conflict of interests.

## AUTHOR CONTRIBUTIONS

Study design and data interpretation: all authors; study conduct: AI, RC; data collection: DK, RJH, JEA, RC; derivation of spine shapes: FRS, AVP, JSG, RJB, RMA; data analysis: AI; drafting manuscript: AI. AI takes responsibility for the integrity of the data analysis. All authors have read and approved the final submitted manuscript.

## Supporting information

Supporting information

Supporting information

## Data Availability

Data used in this publication are available to bona fide researchers upon request to the NSHD Data Sharing Committee via a standard application procedure. Further details can be found at http://www.nshd.mrc.ac.uk/data. http://orcid.org/10.5522/NSHD/Q101; http://orcid.org/10.5522/NSHD/Q102; http://orcid.org/10.5522/NSHD/S102A.
