# Supplementary material for: Motor development in infancy and spine shape in early old age: Findings from a British birth cohort study
Source: J Orthop Res. 2020 Mar 18;38(12):2740–8. doi: 10.1002/jor.24656 (PMC8641380; doi:10.1002/jor.24656)
Supplement: Supplementary file 1 — Supporting information [file JOR-38-2740-s002.docx]

| Milestone | Mode | Group | Model | Regression coefficient | 95% CI | | P | Sex Interaction P |
| --- | --- | --- | --- | --- | --- | --- | --- | --- |
|  |  |  |  |  |  |  |  |  |
| Sitting | SM1 | Combined | 1 | 0.023 | -0.015 | 0.060 | 0.23 | 0.70 |
|  |  |  | 2 | 0.027 | -0.011 | 0.065 | 0.16 | 0.69 |
|  | SM2 | Combined | 1 | -0.017 | -0.055 | 0.021 | 0.37 | 0.42 |
|  |  |  | 2 | -0.025 | -0.063 | 0.014 | 0.21 | 0.42 |
|  | SM3 | Combined | 1 | 0.003 | -0.031 | 0.037 | 0.86 | 0.63 |
|  |  |  | 2 | -0.003 | -0.037 | 0.030 | 0.84 | 0.49 |
|  | SM4 | Combined | 1 | -0.008 | -0.046 | 0.031 | 0.69 | 0.32 |
|  |  |  | 2 | -0.003 | -0.038 | 0.033 | 0.90 | 0.28 |
|  | SM5 | Combined | 1 | -0.031 | -0.069 | 0.006 | 0.10 | 0.39 |
|  |  |  | 2 | -0.036 | -0.075 | 0.002 | 0.06 | 0.42 |
|  | SM6 | Combined | 1 | -0.002 | -0.040 | 0.035 | 0.91 | 0.90 |
|  |  |  | 2 | 0.002 | -0.035 | 0.039 | 0.92 | 0.88 |
|  | SM7 | Combined | 1 | 0.025 | -0.013 | 0.063 | 0.20 | 0.19 |
|  |  |  | 2 | 0.027 | -0.011 | 0.066 | 0.16 | 0.19 |
|  | SM8 | Combined | 1 | -0.008 | -0.044 | 0.029 | 0.69 | 0.60 |
|  |  |  | 2 | -0.002 | -0.038 | 0.035 | 0.94 | 0.48 |
| Standing | SM1 | Combined | 1 | 0.017 | -0.008 | 0.042 | 0.17 | 0.12 |
|  |  |  | 2 | 0.022 | -0.004 | 0.047 | 0.10 | 0.16 |
|  | SM2 | Combined | 1 | -0.002 | -0.027 | 0.023 | 0.87 | 0.25 |
|  |  |  | 2 | -0.012 | -0.037 | 0.014 | 0.37 | 0.30 |
|  | SM3 | Men | 1 | -0.024 | -0.058 | 0.009 | 0.16 | 0.03 |
|  |  | Women |  | 0.027 | -0.002 | 0.056 | 0.07 |  |
|  |  | Men | 2 | -0.039 | -0.073 | -0.005 | 0.02 | 0.01 |
|  |  | Women |  | 0.024 | -0.006 | 0.053 | 0.12 |  |
|  | SM4 | Combined | 1 | -0.002 | -0.028 | 0.024 | 0.88 | 0.13 |
|  |  |  | 2 | 0.005 | -0.022 | 0.031 | 0.74 | 0.14 |
|  | SM5 | Men | 1 | 0.006 | -0.029 | 0.041 | 0.74 | 0.06 |
|  |  | Women |  | -0.046 | -0.083 | -0.008 | 0.02 |  |
|  |  | Men | 2 | -0.002 | -0.038 | 0.034 | 0.91 | 0.09 |
|  |  | Women |  | -0.037 | 0.000 | -0.074 | 0.05 |  |
|  | SM6 | Combined | 1 | 0.028 | 0.003 | 0.052 | 0.03 | 0.54 |
|  |  |  | 2 | 0.027 | 0.002 | 0.052 | 0.04 | 0.53 |
|  | SM7 | Combined | 1 | 0.021 | -0.005 | 0.046 | 0.11 | 0.53 |
|  |  |  | 2 | 0.021 | -0.005 | 0.047 | 0.11 | 0.39 |
|  | SM8 | Combined | 1 | -0.001 | -0.025 | 0.024 | 0.95 | 0.16 |
|  |  |  | 2 | 0.008 | -0.017 | 0.032 | 0.55 | 0.21 |

Supplementary Table 1. Associations between age at onset of independent sitting and standing, and spine shape mode outcomes in the MRC National Survey of Health and Development. Regression coefficients are the difference in mean SM scores per 1 month increase in sitting or standing age. Where sex interactions were evident (*P* for interaction < 0.1), sex-specific associations are presented.

Footnote: Model 1 adjusted for Sex (if men and women are combined) and Clinic, Model 2: Model 1 + Birthweight + Father’s occupational Class + Adult Occupational Class + Height + Appendicular Fat Mass + Appendicular Lean Mass.
