# Supplementary material for: Motor development in infancy and spine shape in early old age: Findings from a British birth cohort study
Source: J Orthop Res. 2020 Mar 18;38(12):2740–8. doi: 10.1002/jor.24656 (PMC8641380; doi:10.1002/jor.24656)
Supplement: Supplementary file 2 — Supporting information [file JOR-38-2740-s001.docx]

**Supplementary Figure 1. Descriptions and illustrations of key features of spine mode scores SM1-SM8**

| Mode  (%Var) | Description | ±2 SD overlay | -2 SD +2 SD |
| --- | --- | --- | --- |
| 1  (53.0%) | **Curviness**  Total amount and direction of curvature within the spine from L5 to T10.  Negative scores:   - Flatter lumbar lordosis and a slight kyphosis in thoracic region (T12-T10).   Positive scores:   - Overall greater curvature throughout, increasing lordosis in both lumbar and thoracic sections. | 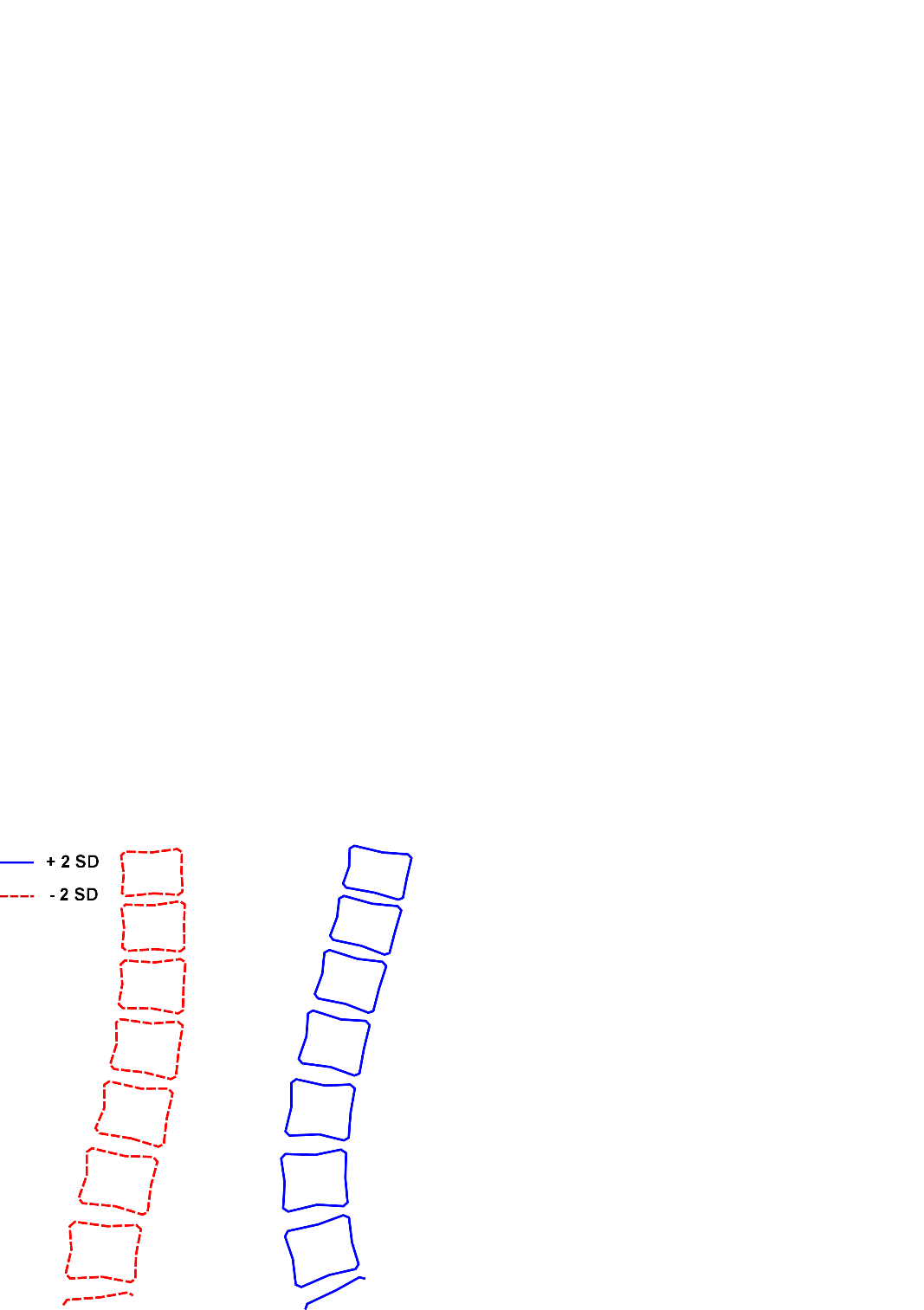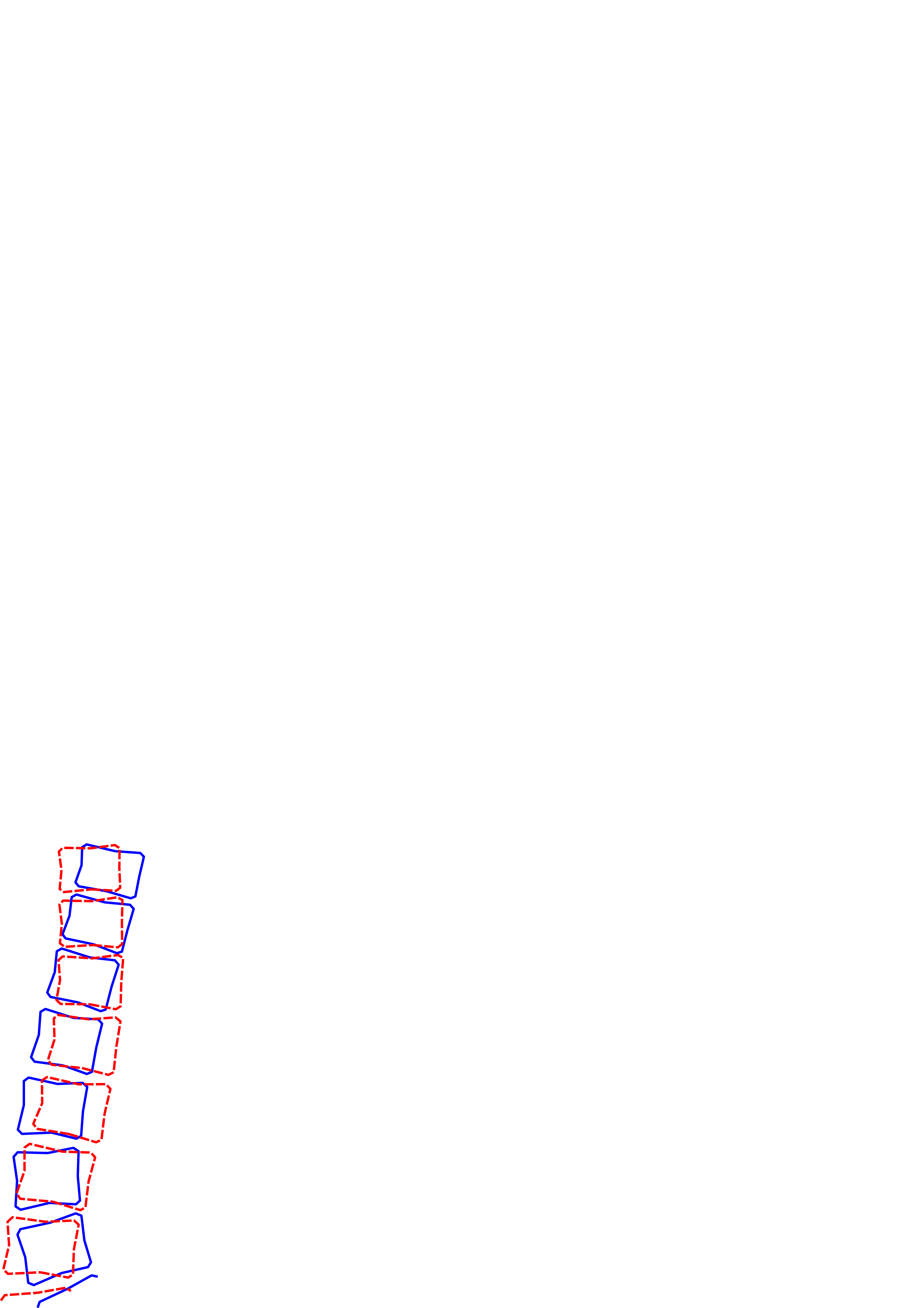 | 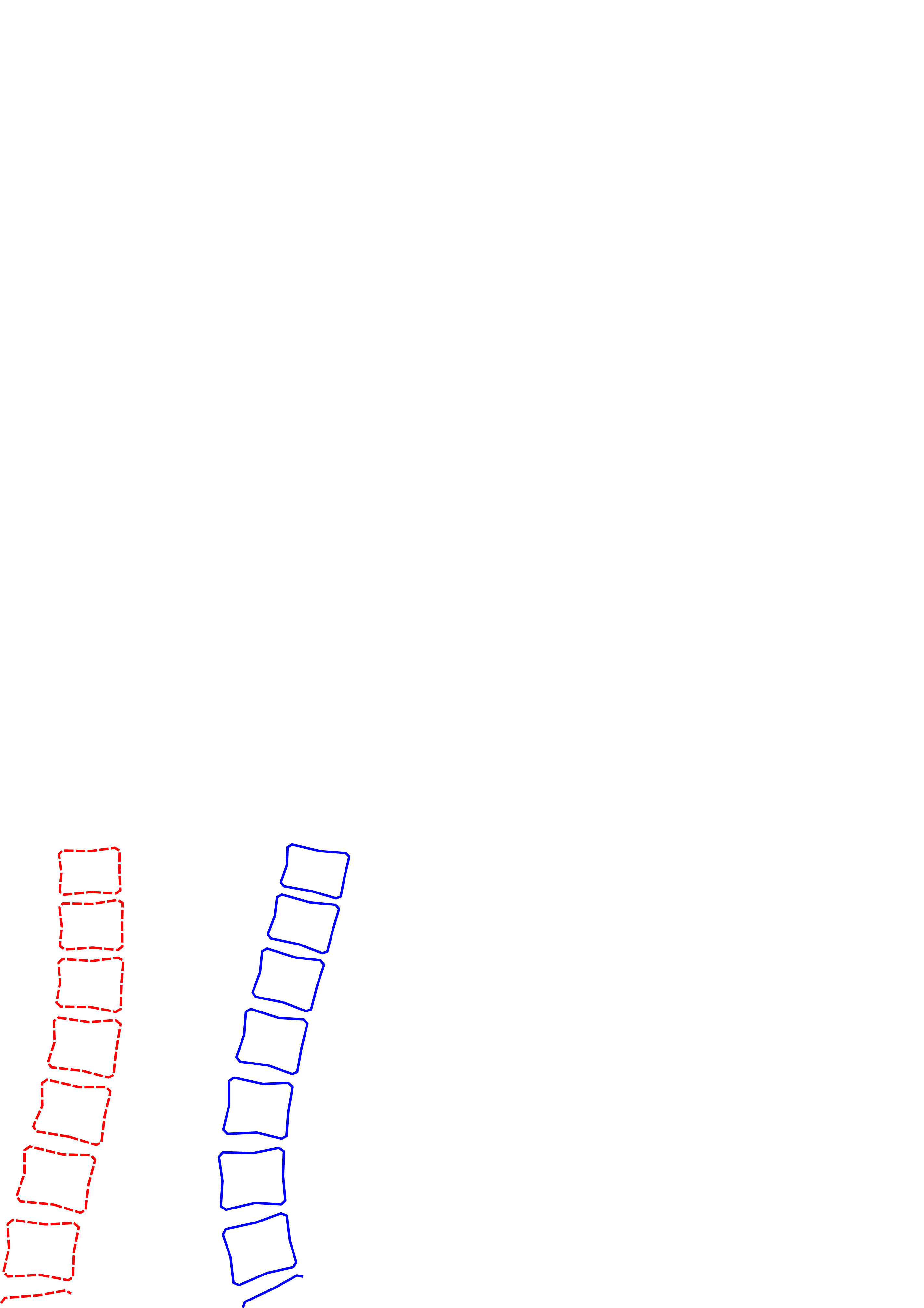  T10  L5 |
| 2  (10.0%) | **Evenness**  Differences in the distribution of curvature along the length of the spine, with consequent small variations in disc space.  Negative scores:   - Snaking curve with a lumbar lordosis and thoracic kyphosis centred around L1/T12.   Positive scores:   - Increasingly evenly distributed curvature throughout all sections from L5-T10 (superimposed on lordotic, straight or kyphotic overall shape described by SM1). | 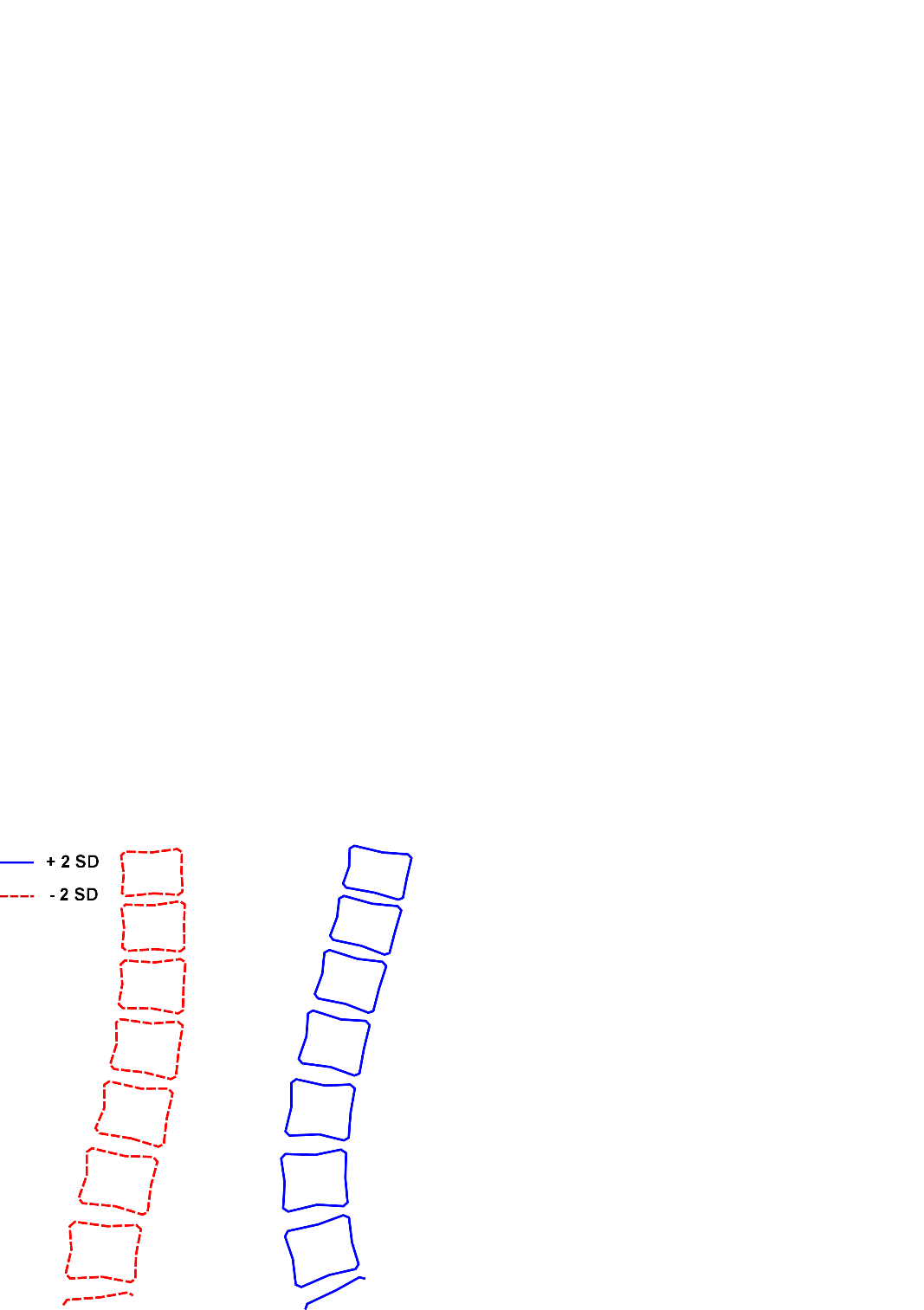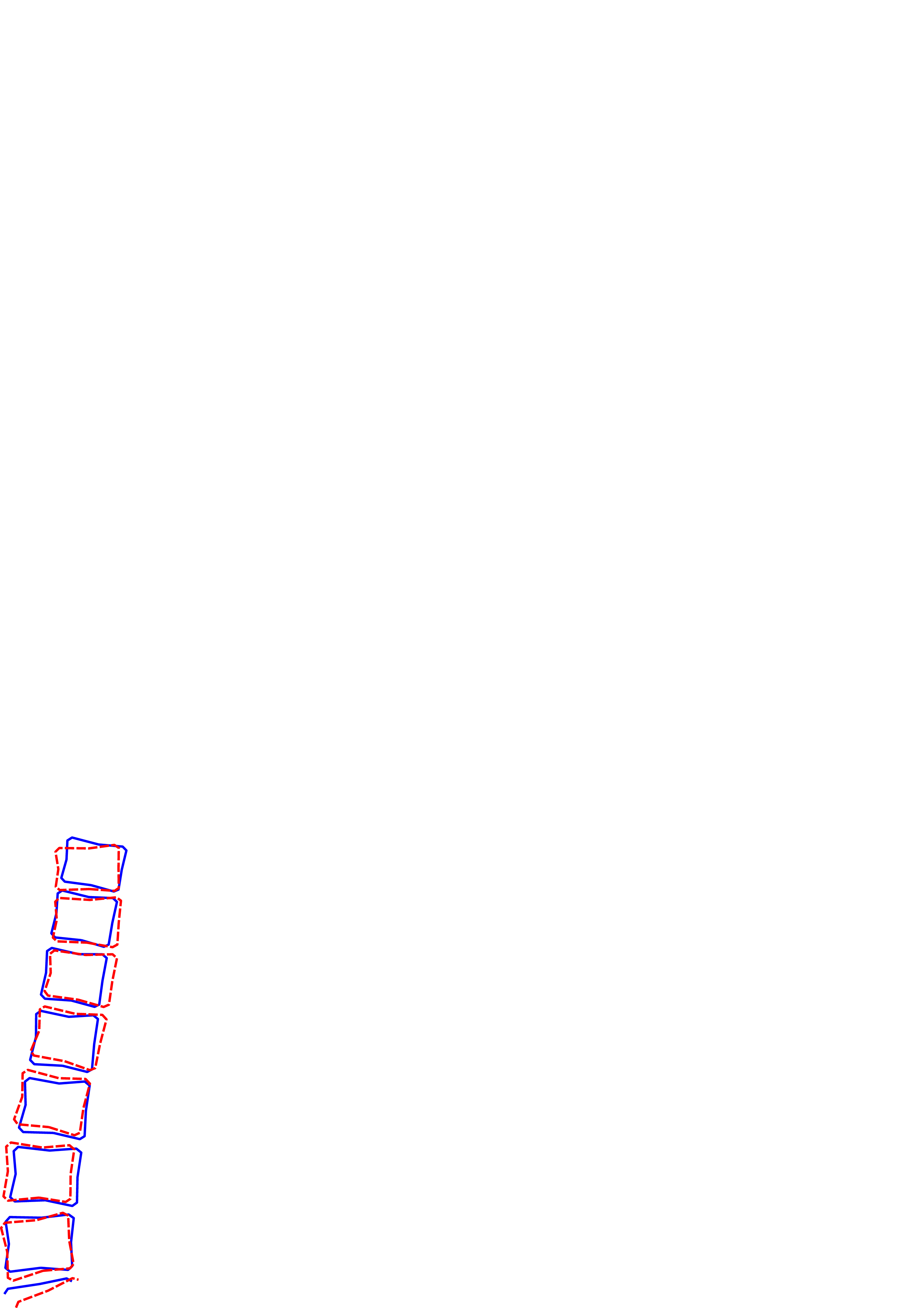 | 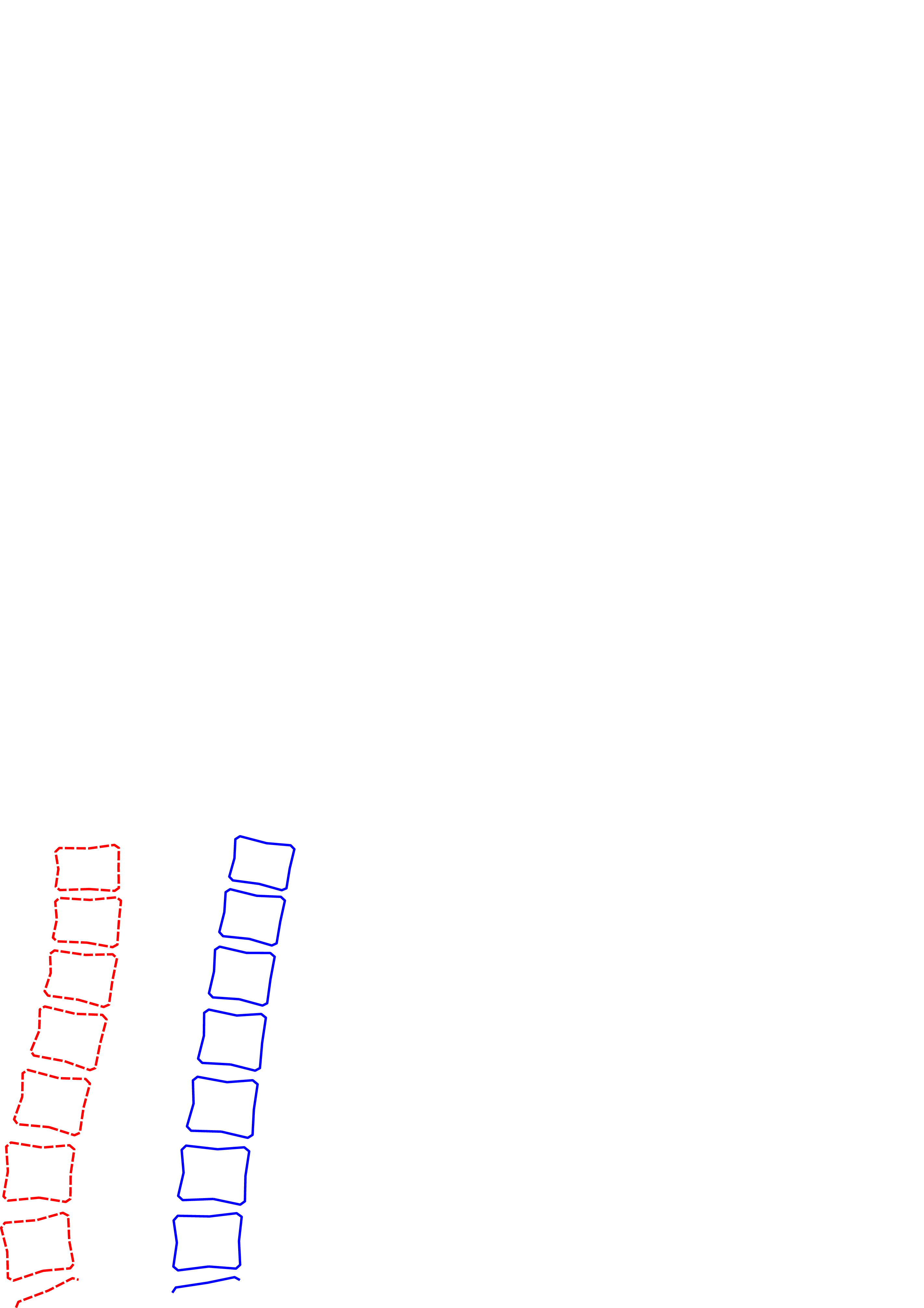 |
| 3  (8.6%) | Relative anterior-posterior diameter  Negative scores:   - Greater relative vertebral body a-p diameter   Positive scores:   - Smaller relative vertebral body a-p diameter. | 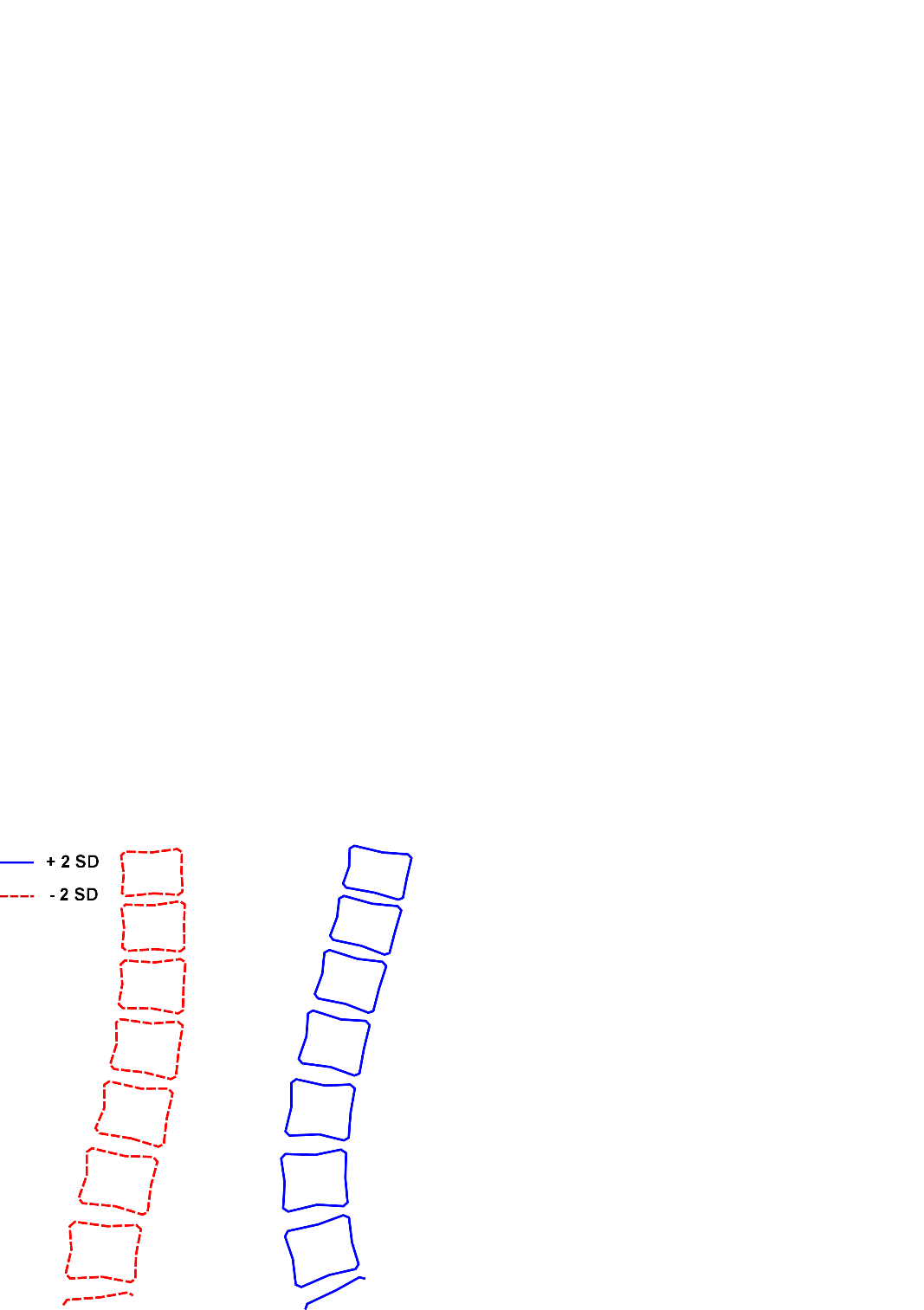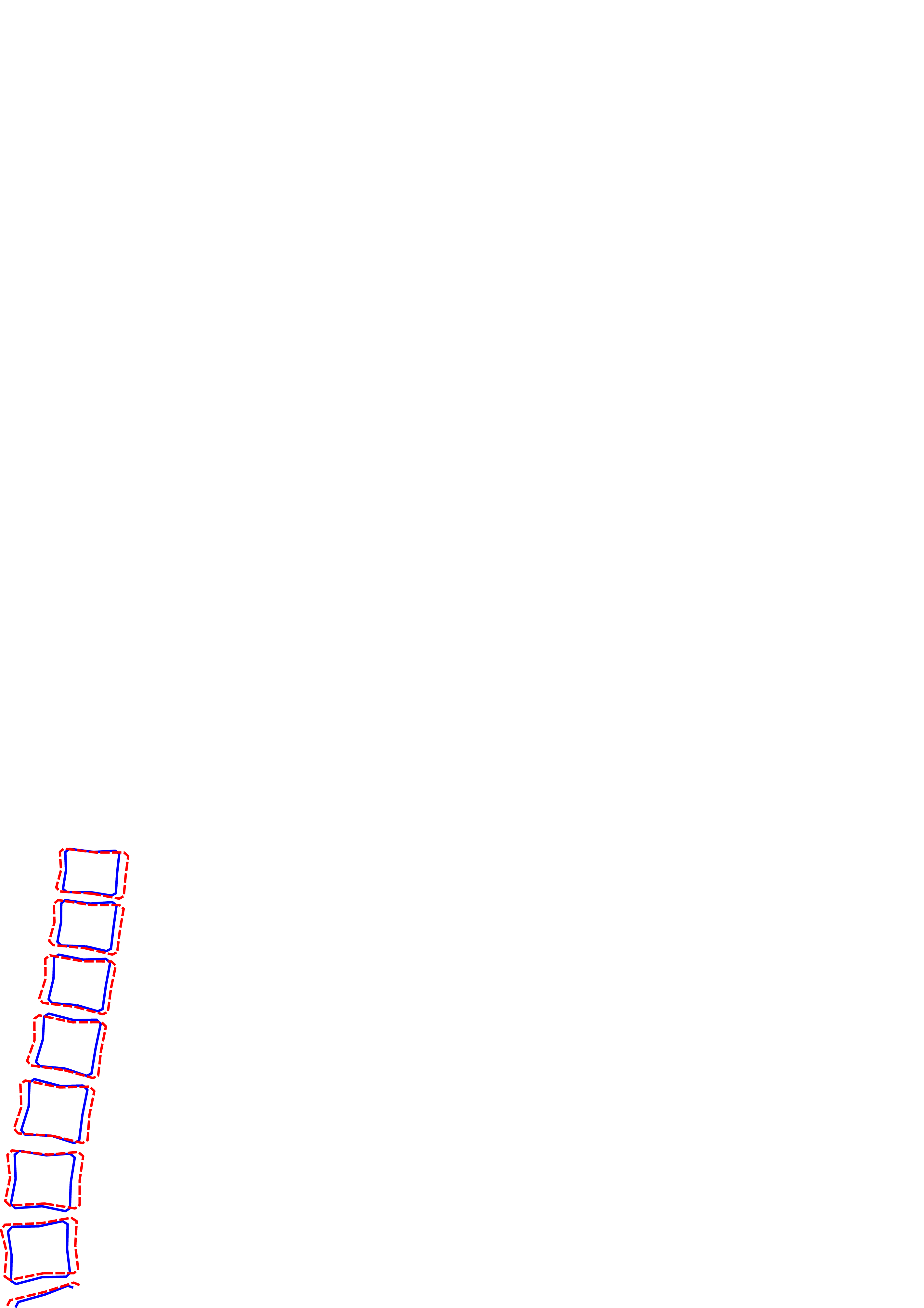 | 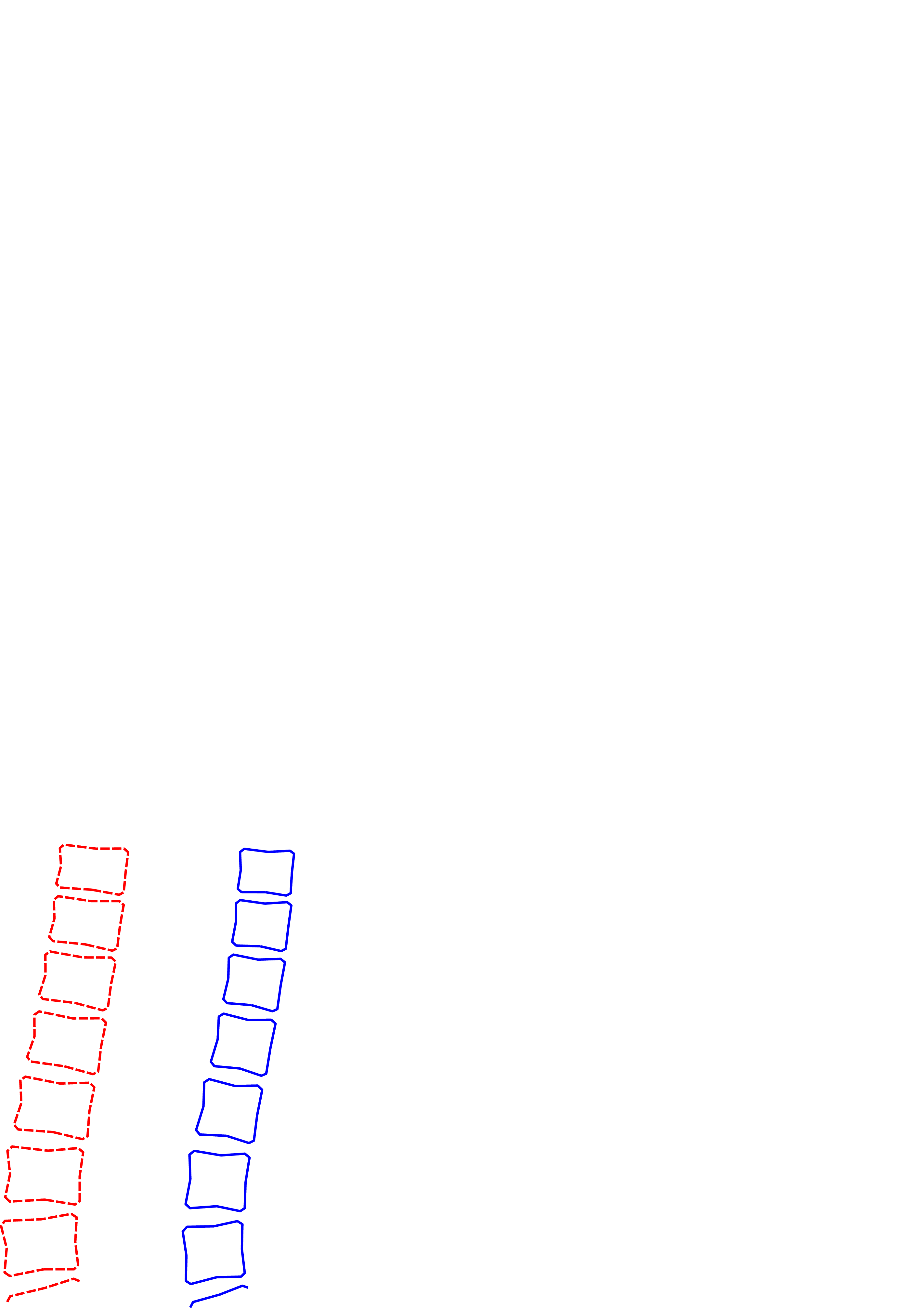 |
| 4  (7.1%) | A combination of vertebral rotation at L5-L4 and T10 together with changes in disc space  Negative scores:   - Minor snaking of the curvature with greater anti-clockwise rotation at L5, L4, and T10 with smaller caudal disc spaces.   Positive scores:   - More uniform shape with smaller cranial disc spaces. | 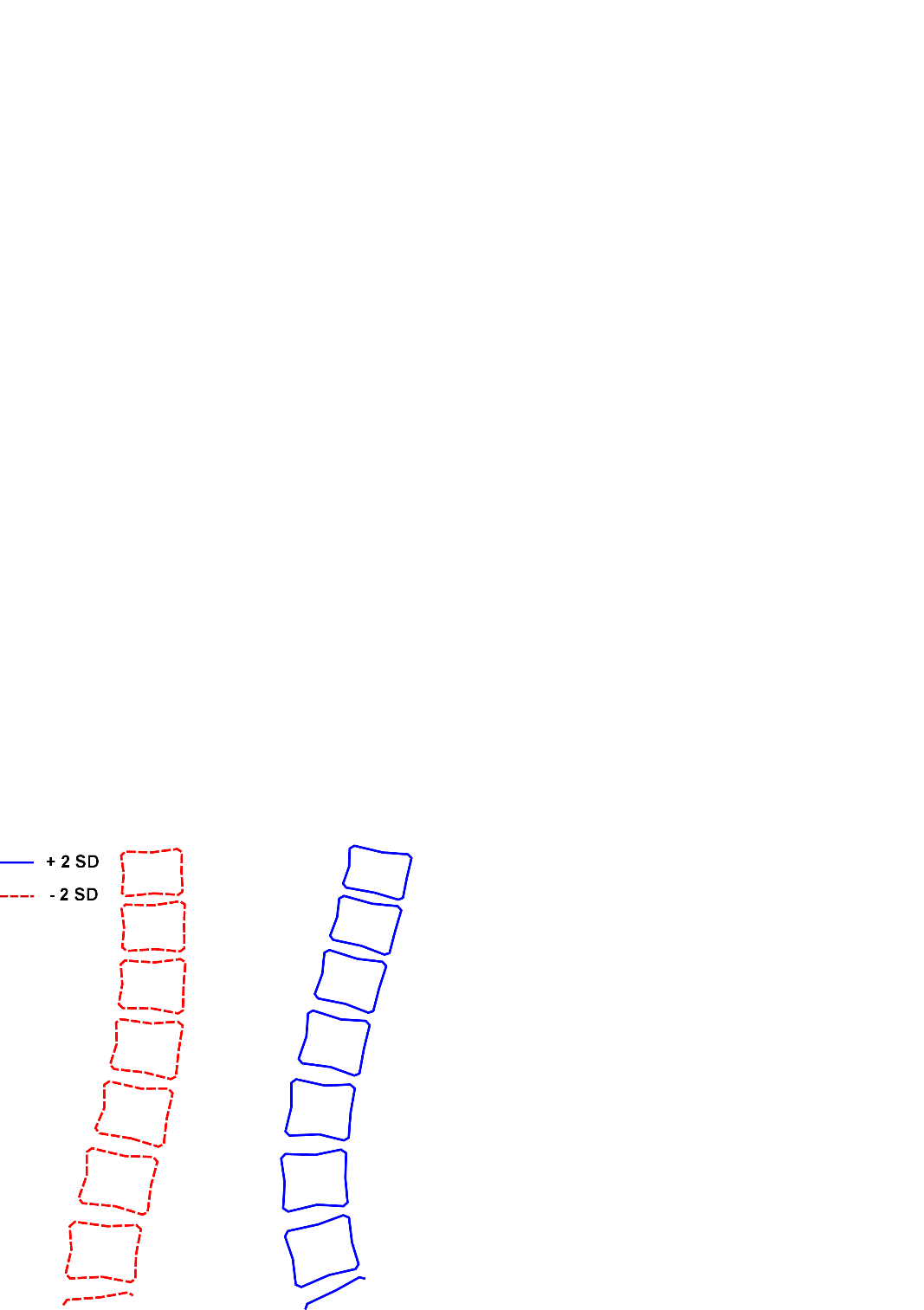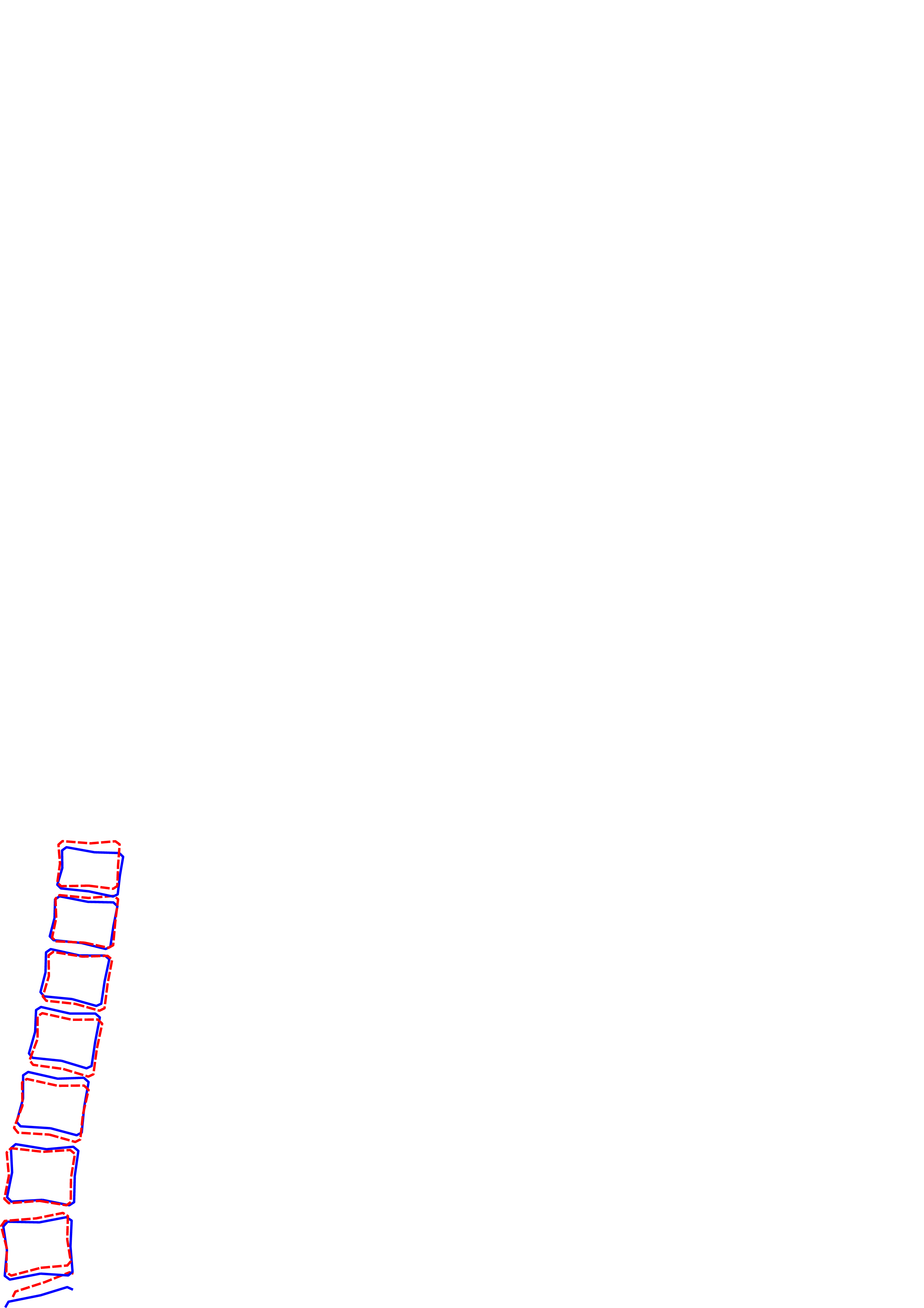 | 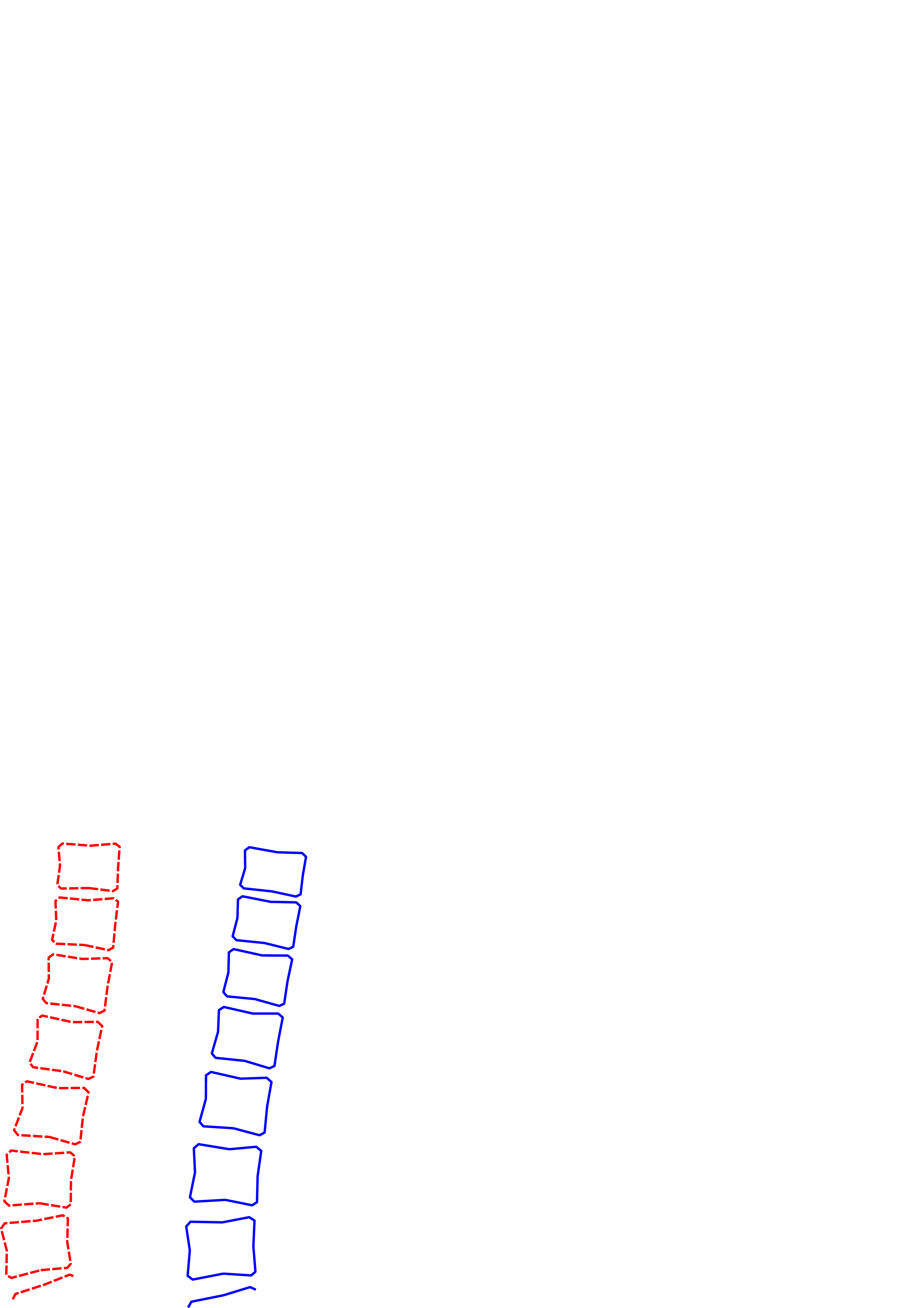 |
| 5  (2.1%) | Vertebral rotation at T10, L3 and L5 with resulting minor variations of lordosis and kyphosis and varying L4/L5 disc space.  Negative scores:   - Thoracic section tending towards a kyphosis but a flatter lordosis (anti-clockwise rotation at T10 and L3, clockwise rotation at L5). - Smaller L5 anterior tilt and narrower L4/L5 disc space.   Positive scores:   - straighter T10-L2 section with a slightly greater lordosis from L3-L5 (clockwise rotation at T10 and L3, anti-clockwise rotation at L5). - Greater L5 anterior tilt and wider L4/L5 disc space. | 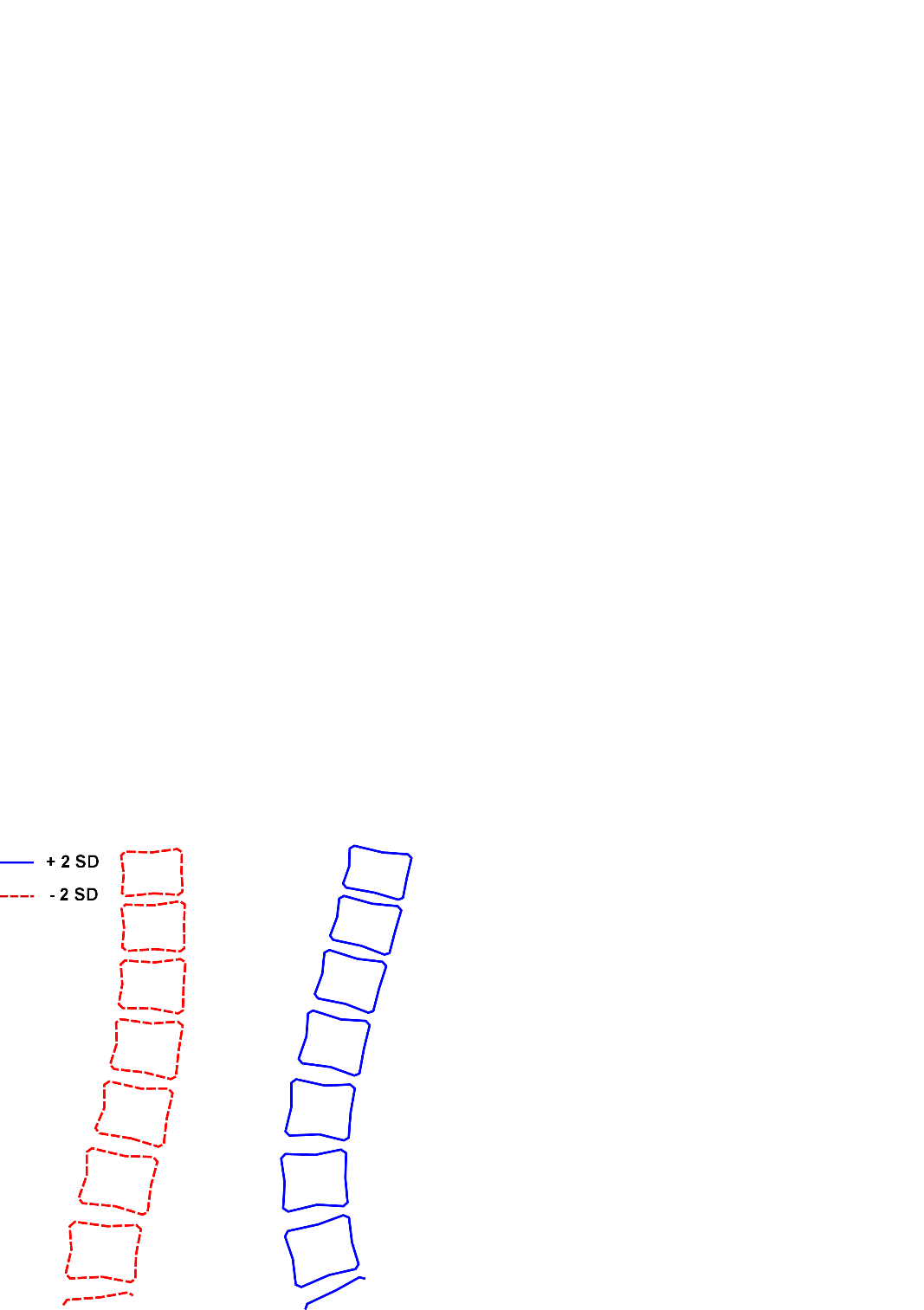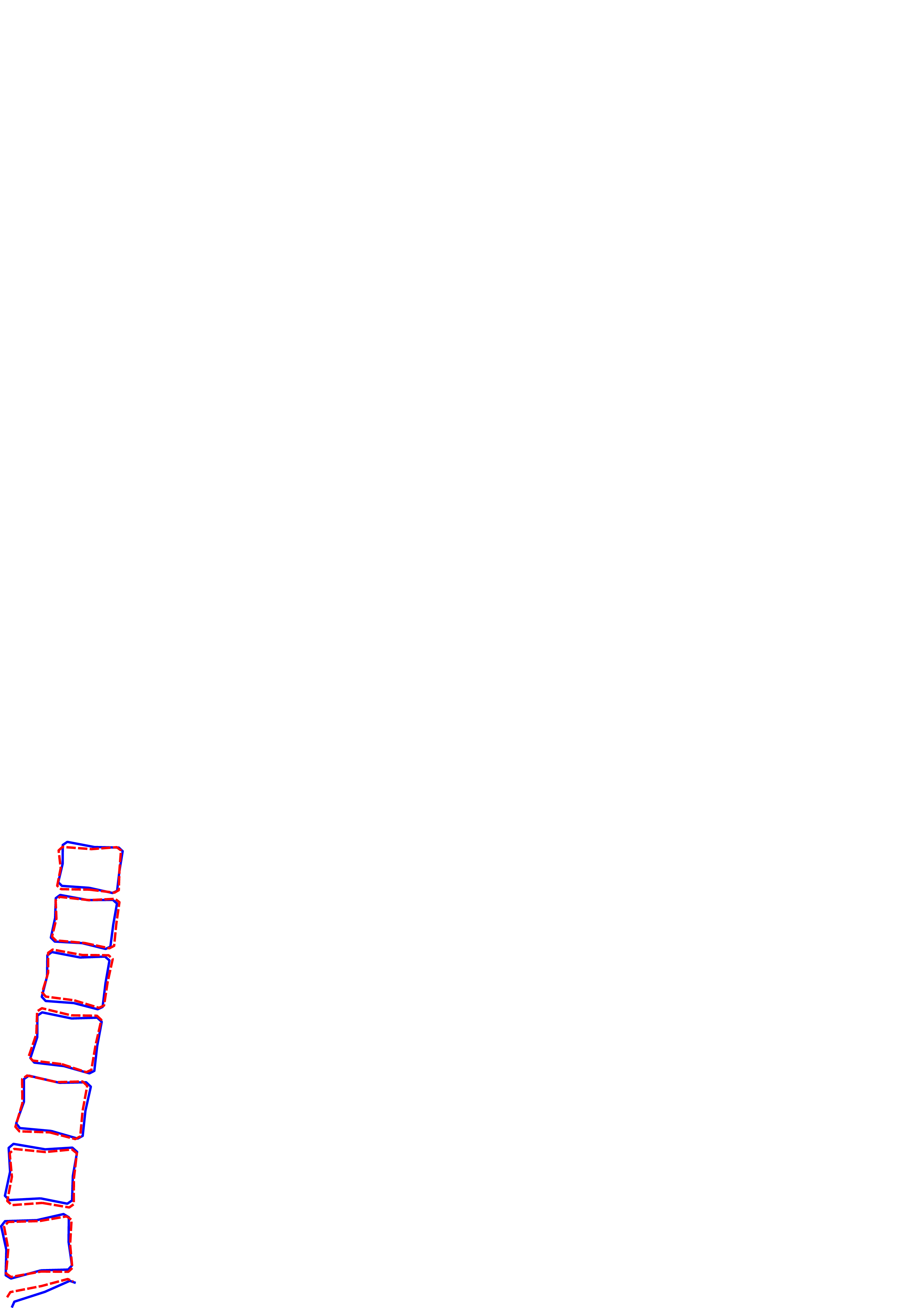 | 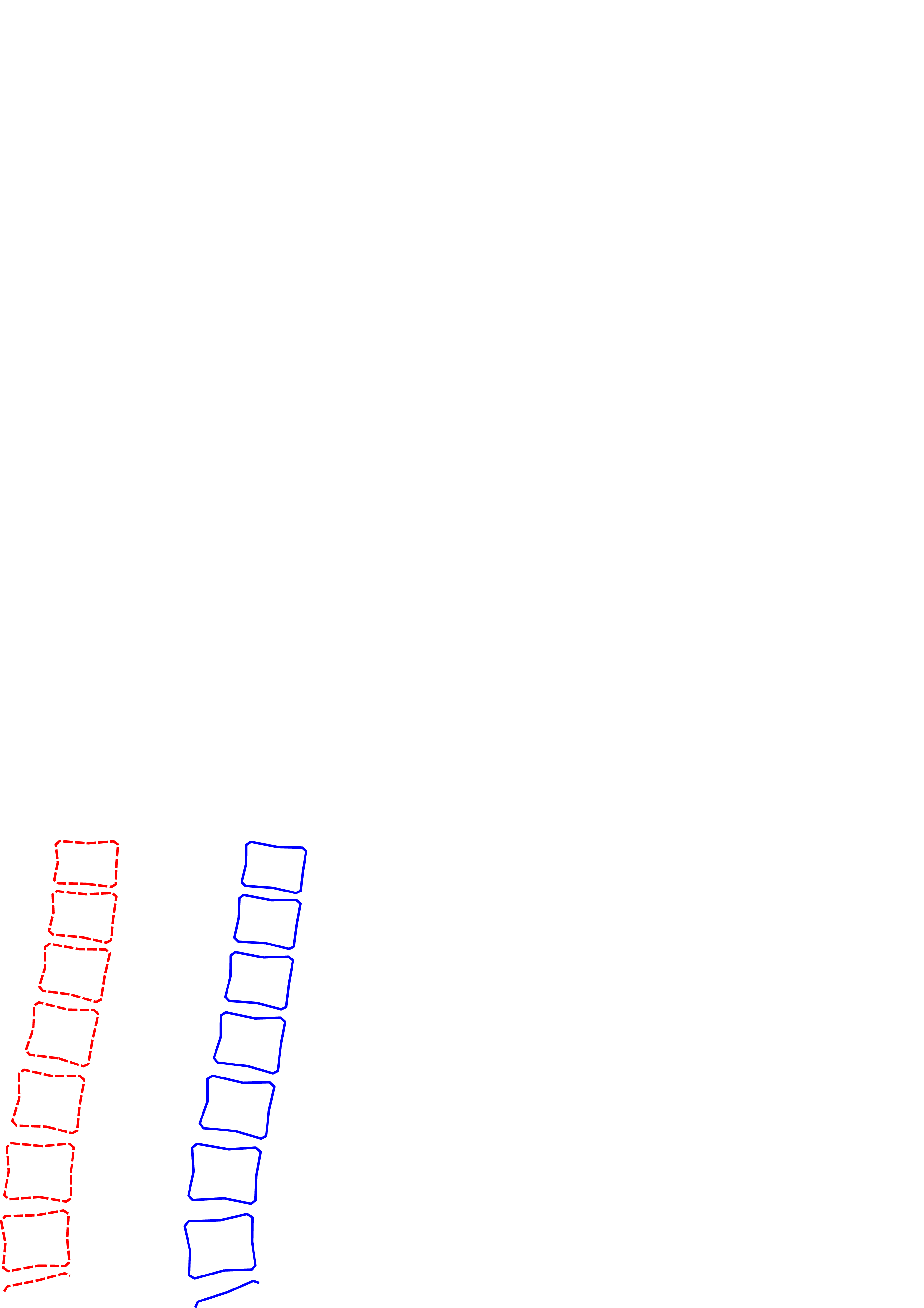 |
| 6  (1.5%) | Difference in anteroposterior vertebral body diameter cranially to caudally.  Negative scores:   - Smaller a-p diameters cranially; smaller than average at T10, T11 but wider than average at L3-L5. - Smaller L4/L5 disc space.   Positive scores:   - More uniform a-p diameters; greater than average at T10, T11 but smaller than average at L3-L5. - Greater L4/L5 disc space. | 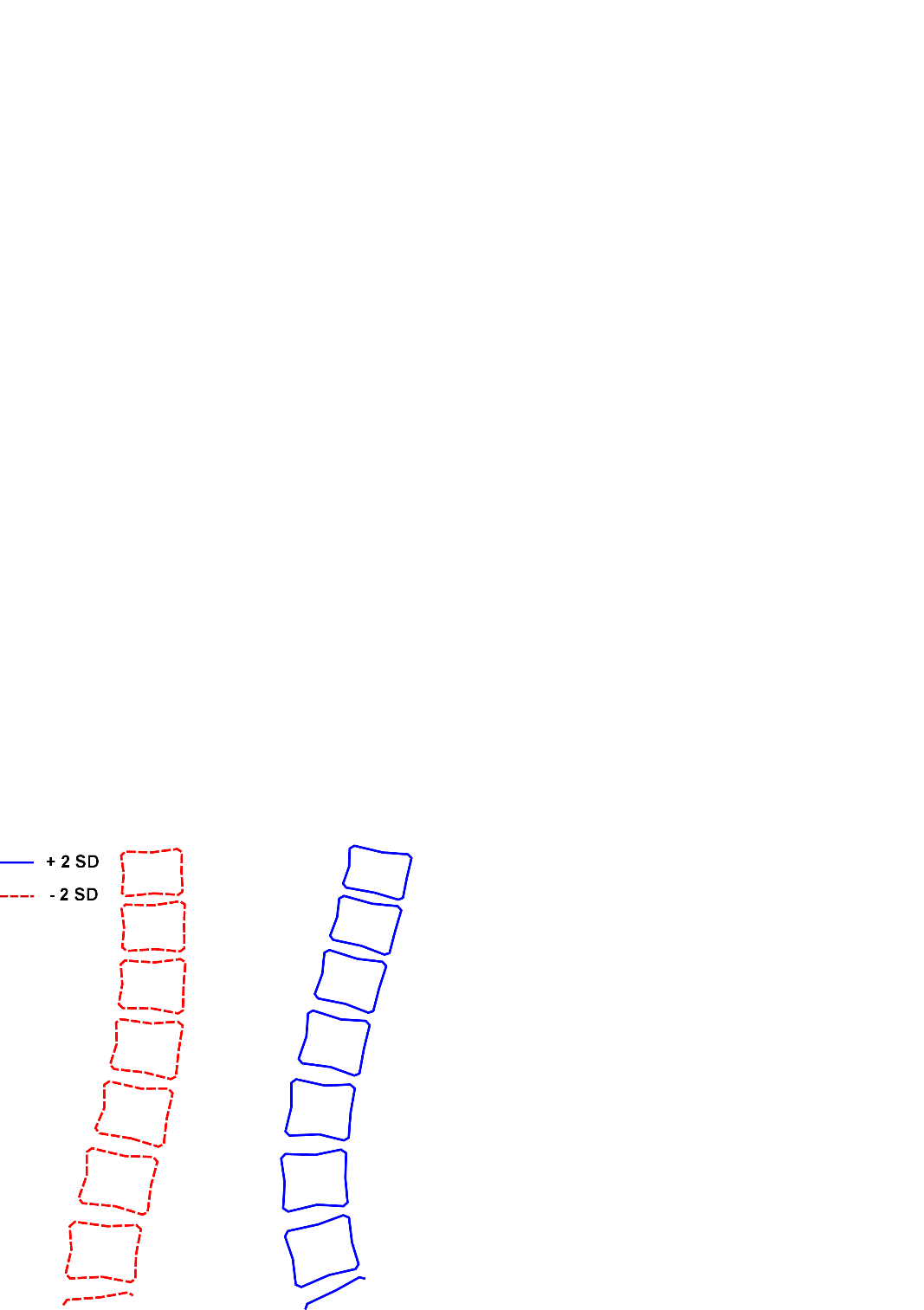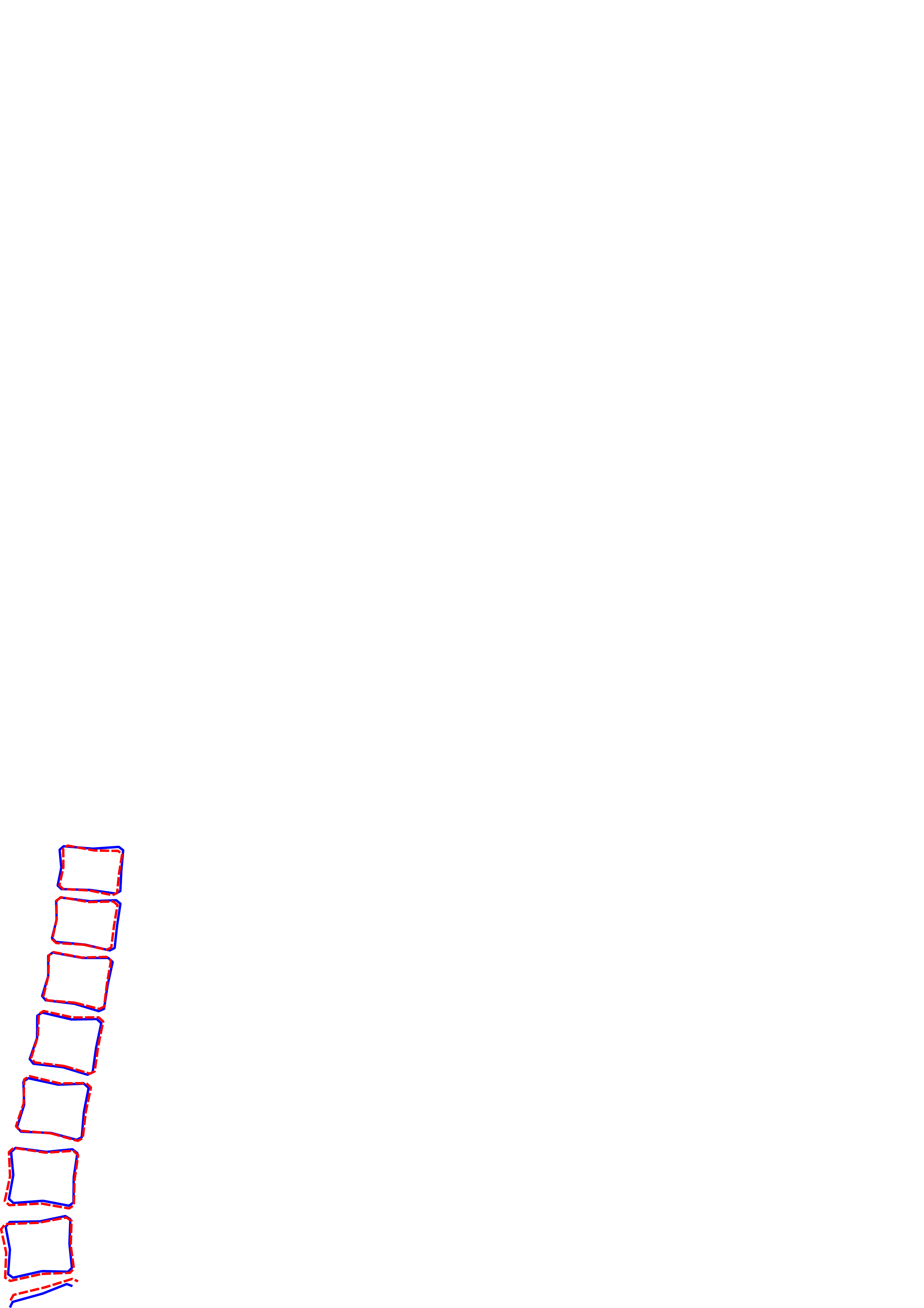 | 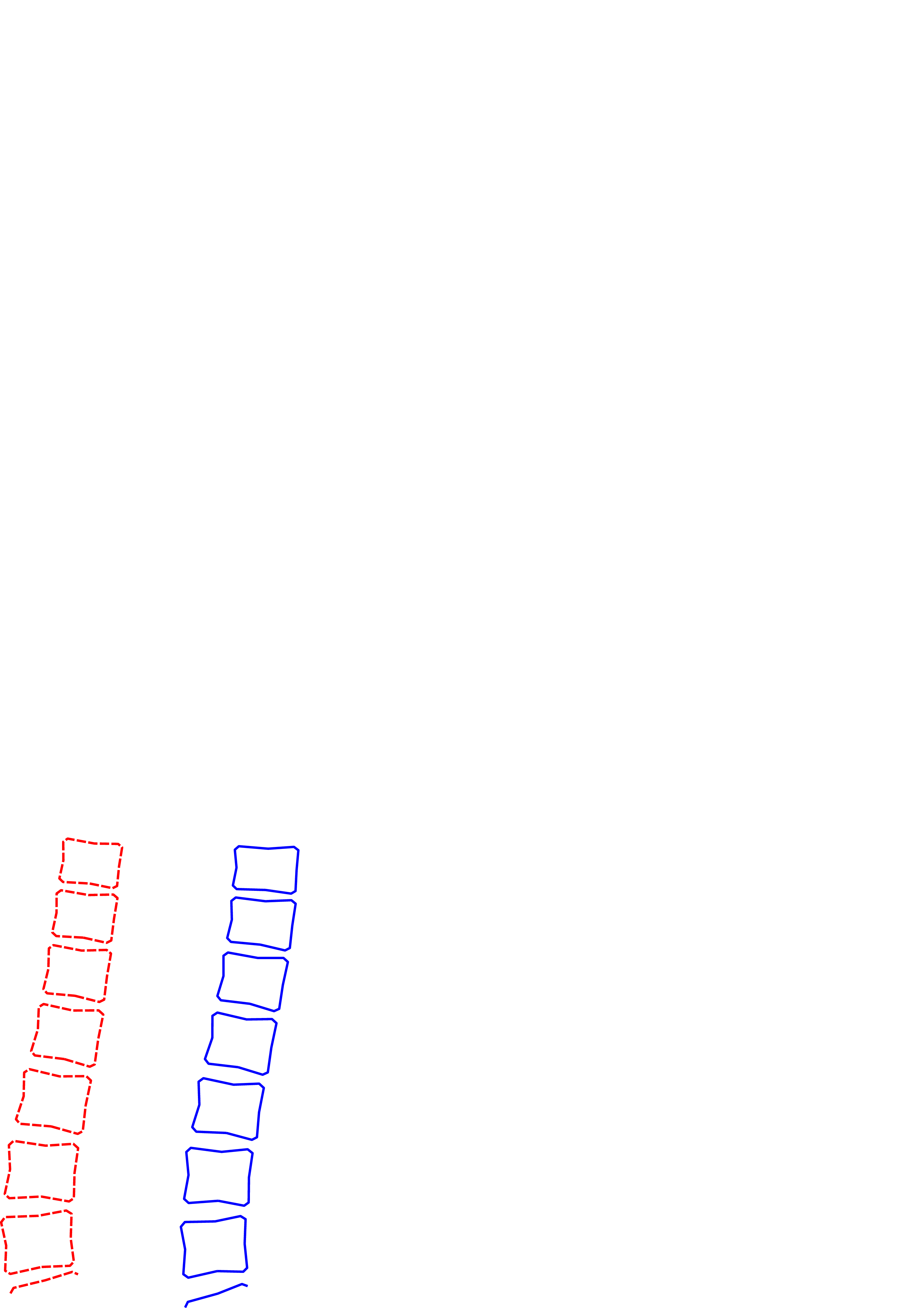 |
| 7  (1.3%) | Minor variation in a-p diameter at T10-T12 and at L4-L5  Negative scores:   - Smaller T10-T12, but larger than average L4-L5 a-p diameter. Squarer vertebral bodies in thoracic section.   Positive scores:   - Greater T10-T12 but smaller than average L4-L5 a-p diameter. | 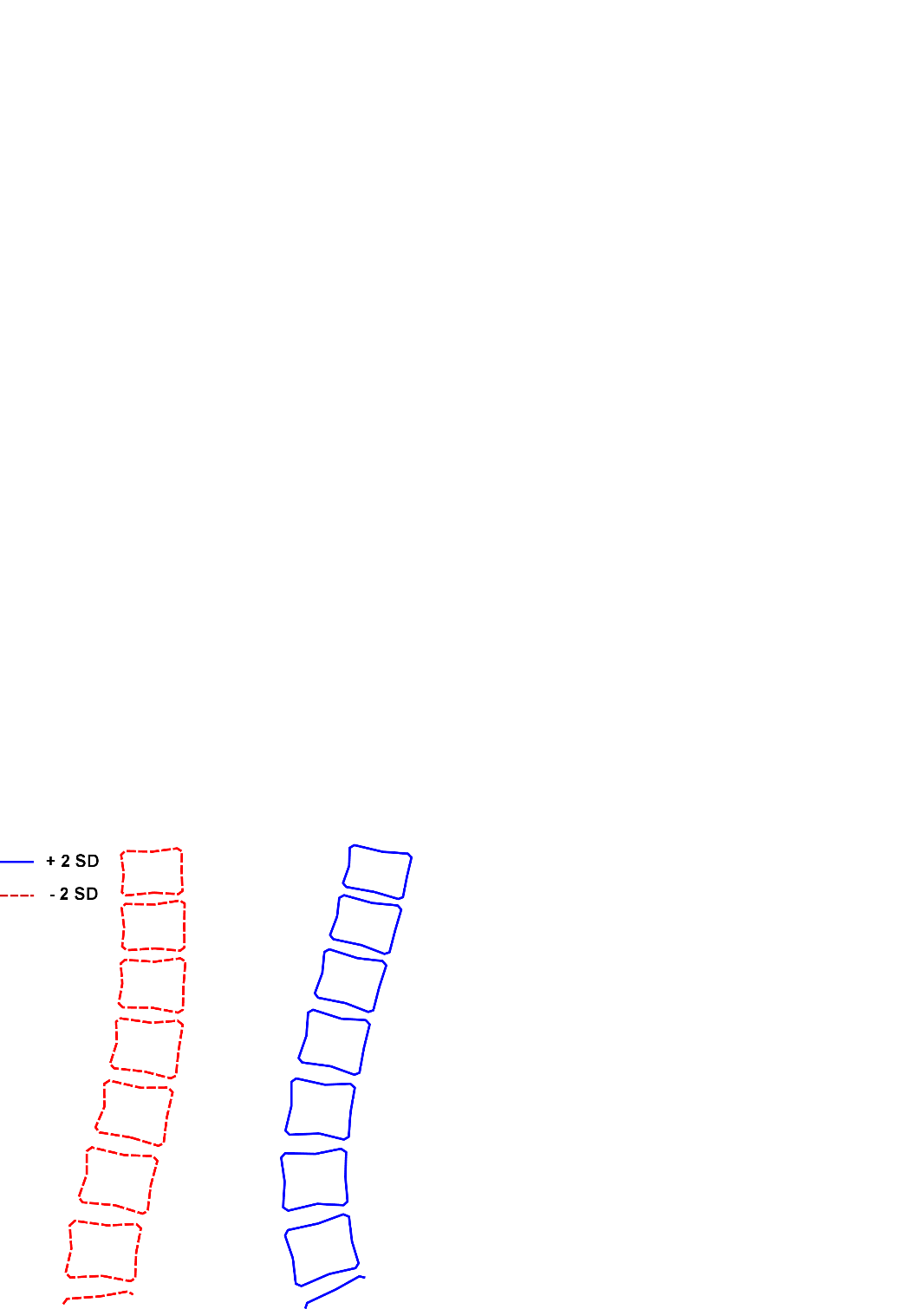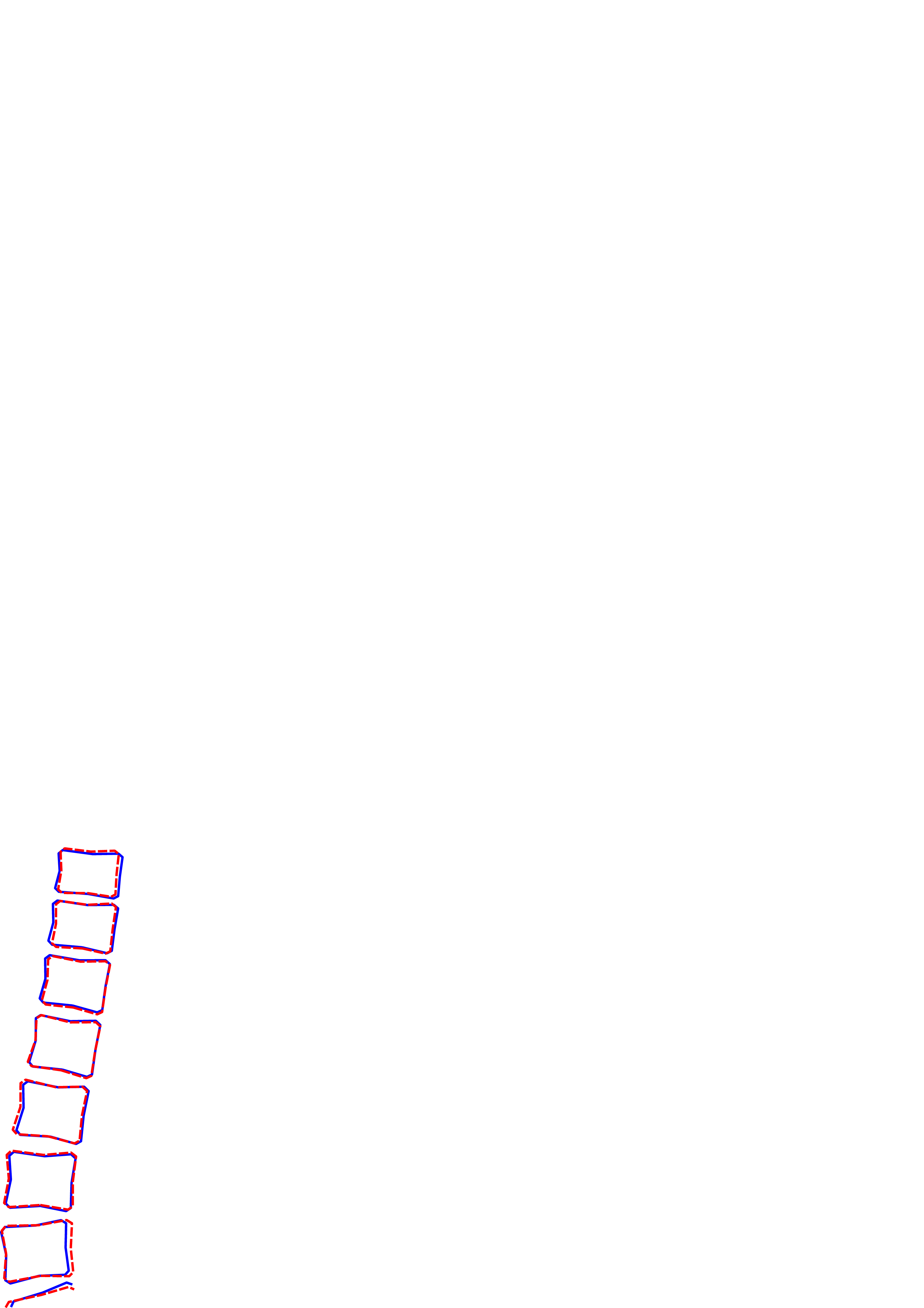 | 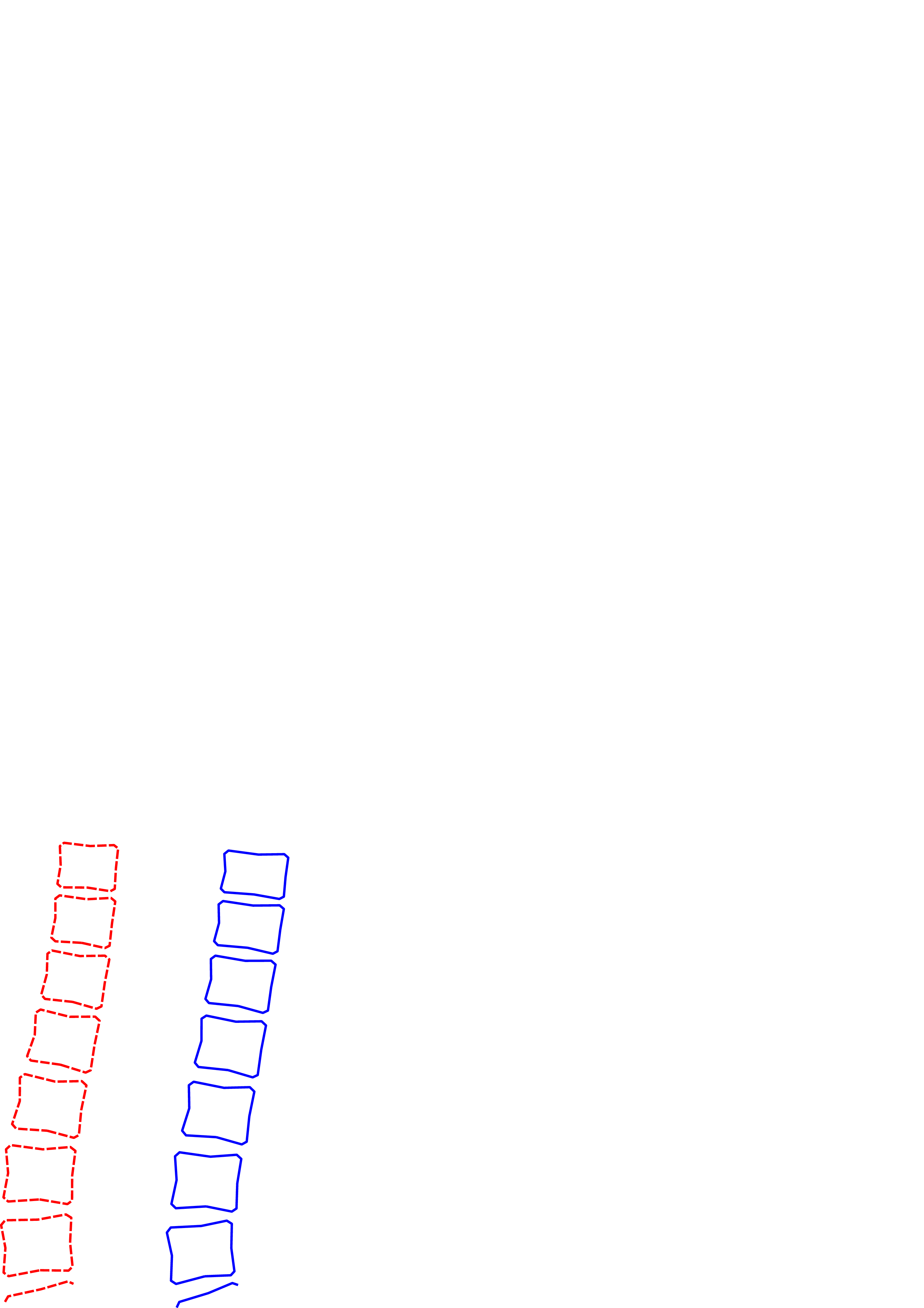 |
| 8  (1.2%) | Variation in L2-L4 vertebral body height, with consequent variation in disc space.  Negative scores:   - Smaller vertebral body heights, relatively larger disc spaces.   Positive scores:   - Taller vertebral body heights, relatively smaller disc spaces. | 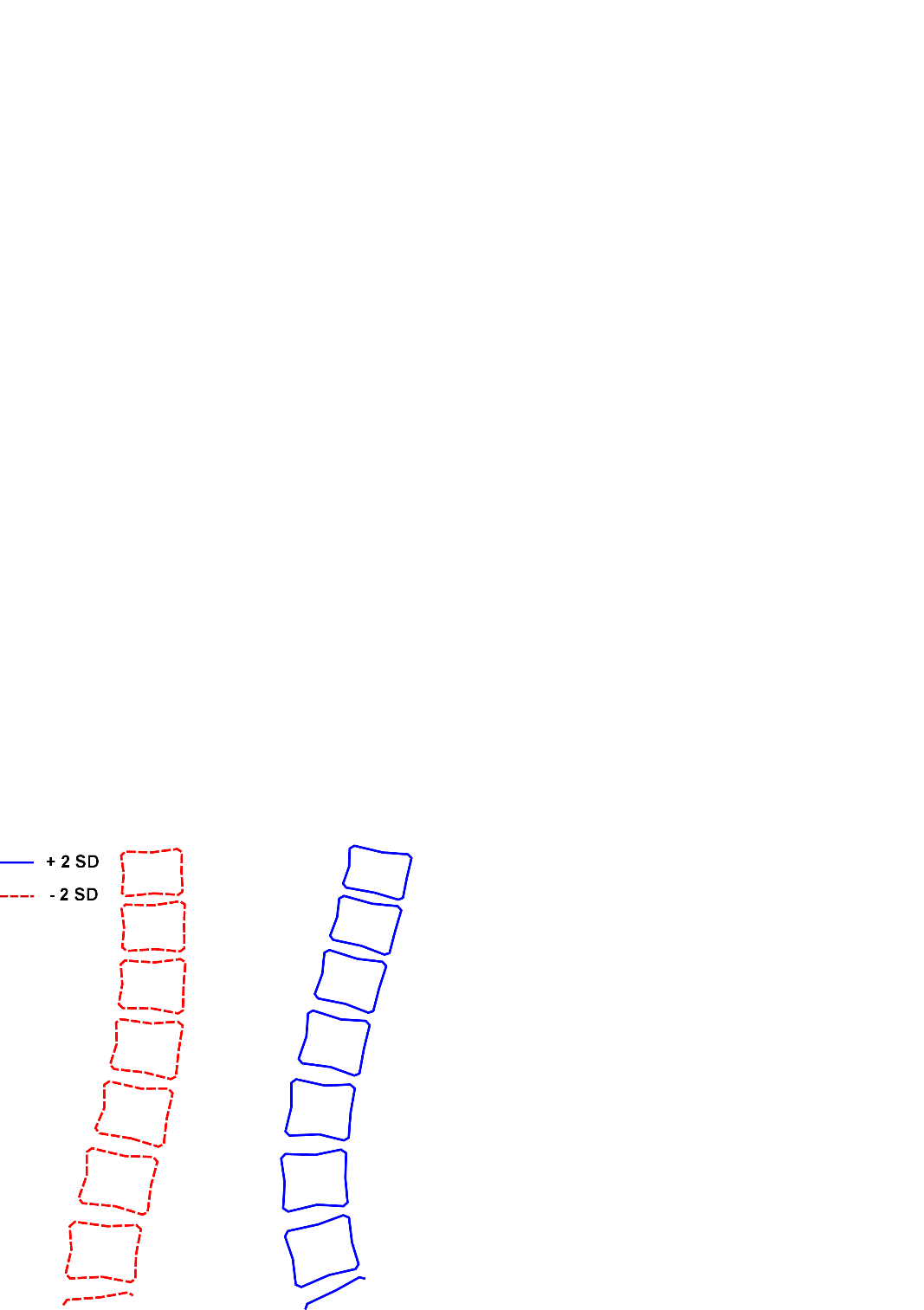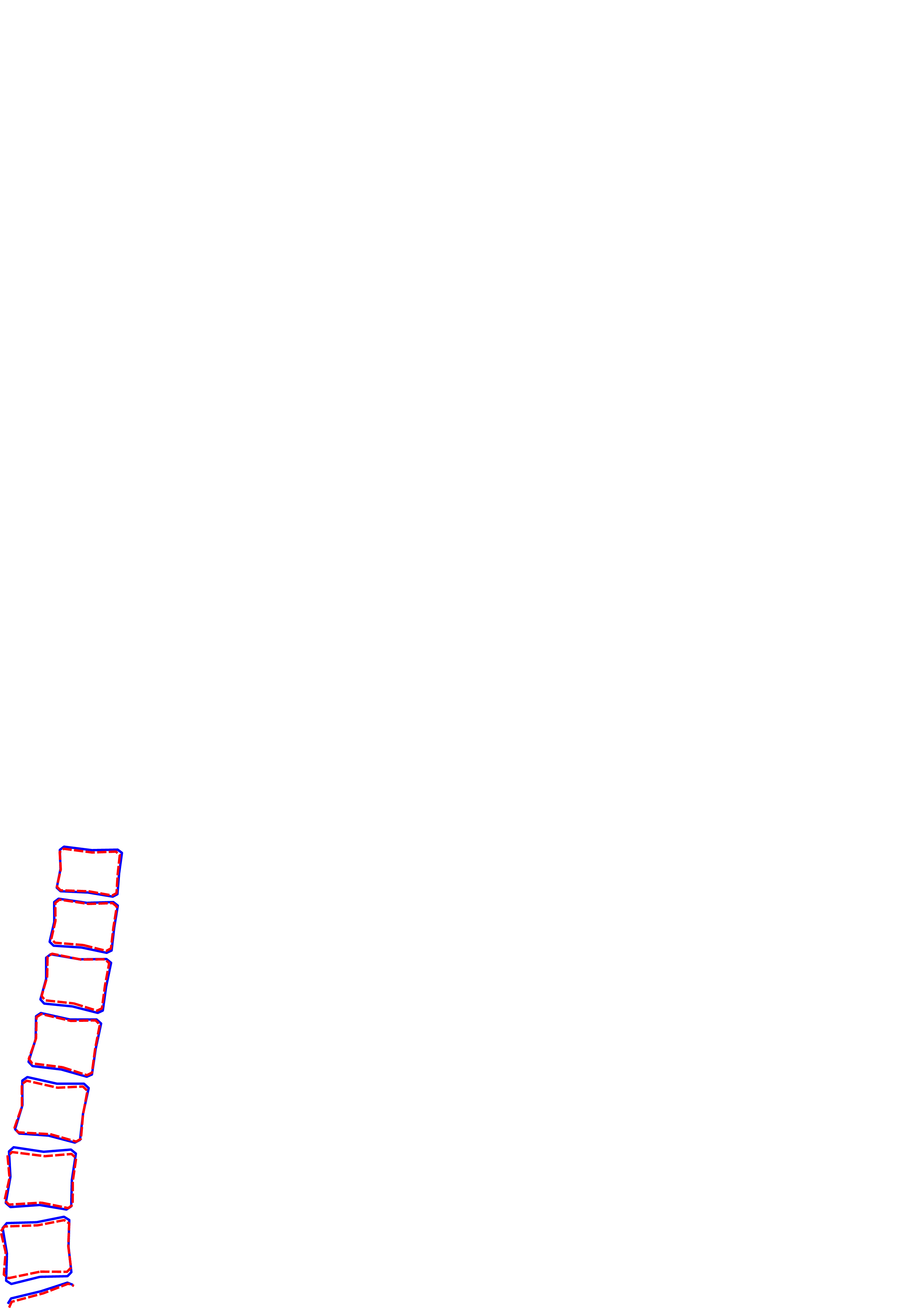 | 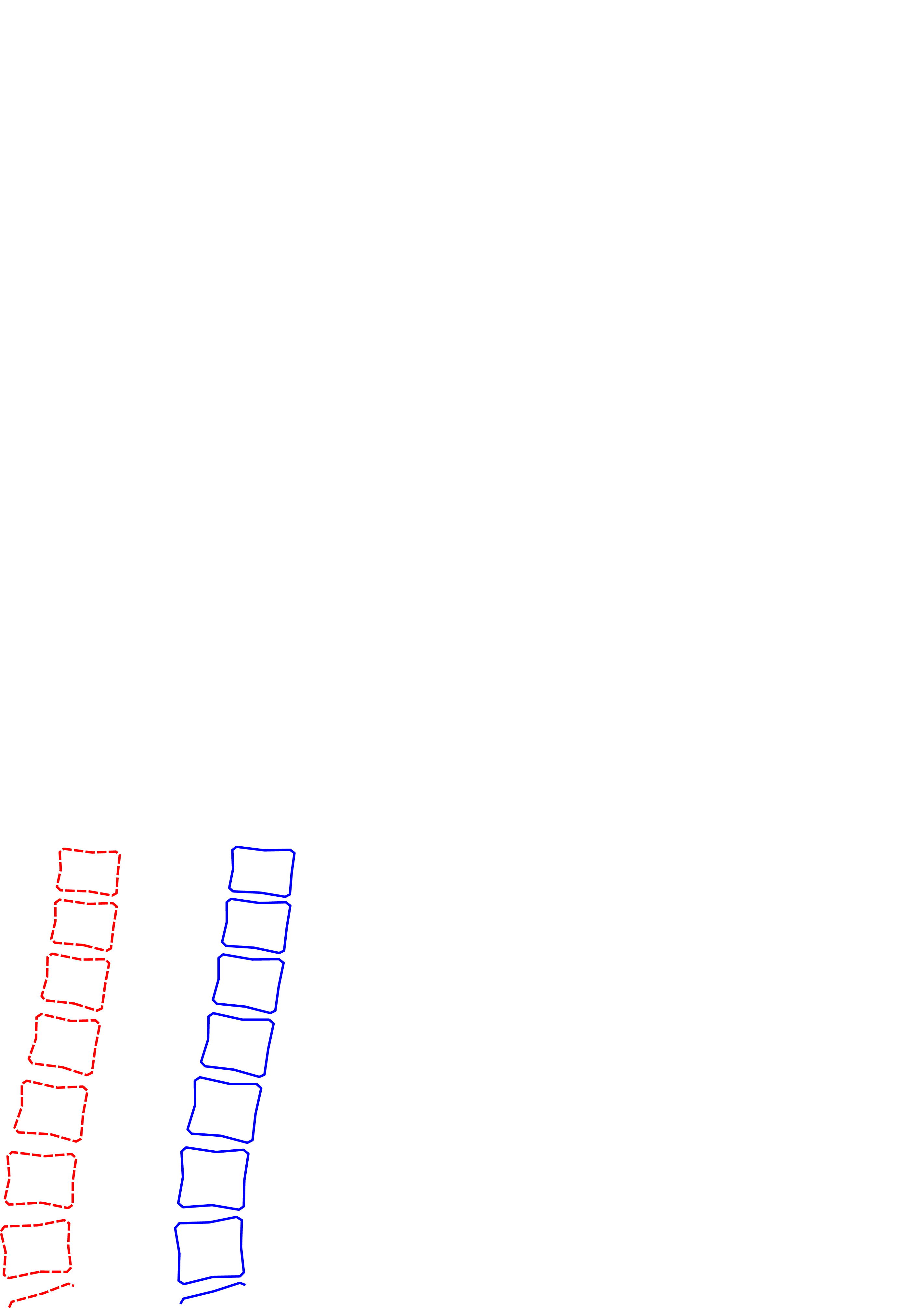 |

This table was originally published in Journal of Anatomy 2017 231(2) pp248-259 <https://doi.org/10.1111/joa.12631> Statistical shape modelling of hip and lumbar spine morphology and their relationship in the MRC National Survey of Health and Development, Anastasia V. Pavlova, Fiona R. Saunders, Stella G. Muthuri, Jennifer S. Gregory, [Rebecca J. Barr](https://onlinelibrary.wiley.com/action/doSearch?ContribAuthorStored=Barr%2C+Rebecca+J), [Kathryn R. Martin](https://onlinelibrary.wiley.com/action/doSearch?ContribAuthorStored=Martin%2C+Kathryn+R), [Rebecca J. Hardy](https://onlinelibrary.wiley.com/action/doSearch?ContribAuthorStored=Hardy%2C+Rebecca+J), [Rachel Cooper](https://onlinelibrary.wiley.com/action/doSearch?ContribAuthorStored=Cooper%2C+Rachel), [Judith E. Adams](https://onlinelibrary.wiley.com/action/doSearch?ContribAuthorStored=Adams%2C+Judith+E), Diana Kuh & Richard M. Aspden, as a full open access paper (Creative commons licence CC BY).
